# Skeletal Remains from Punic Carthage Do Not Support Systematic Sacrifice of Infants

**DOI:** 10.1371/journal.pone.0009177

**Published:** 2010-02-17

**Authors:** Jeffrey H. Schwartz, Frank Houghton, Roberto Macchiarelli, Luca Bondioli

**Affiliations:** 1 Department of Anthropology, University of Pittsburgh, Pittsburgh, Pennsylvania, United States of America; 2 History and Philosophy of Science, University of Pittsburgh, Pittsburgh, Pennsylvania, United States of America; 3 Veterans Research Foundation, Pittsburgh, Pennsylvania, United States of America; 4 Département de Préhistoire, UMR 7194 Centre National de la Recherche Scientifique (CNRS), Muséum national d'Histoire naturelle, Paris, France; 5 Département Géosciences, Université de Poitiers, Poitiers, France; 6 Sezione di Antropologia, Museo Nazionale Preistorico Etnografico “L. Pigorini”, Roma, Italia; University of Delaware, United States of America

## Abstract

Two types of cemeteries occur at Punic Carthage and other Carthaginian settlements: one centrally situated housing the remains of older children through adults, and another at the periphery of the settlement (the “Tophet”) yielding small urns containing the cremated skeletal remains of very young animals and humans, sometimes comingled. Although the absence of the youngest humans at the primary cemeteries is unusual and worthy of discussion, debate has focused on the significance of Tophets, especially at Carthage, as burial grounds for the young. One interpretation, based on two supposed eye-witness reports of large-scale Carthaginian infant sacrifice [Kleitarchos (3^rd^ c. BCE) and Diodorus Siculus (1^st^ c. BCE)], a particular translation of inscriptions on some burial monuments, and the argument that if the animals had been sacrificed so too were the humans, is that Tophets represent burial grounds reserved for sacrificial victims. An alternative hypothesis acknowledges that while the Carthaginians may have occasionally sacrificed humans, as did their contemporaries, the extreme youth of Tophet individuals suggests these cemeteries were not only for the sacrificed, but also for the very young, however they died. Here we present the first rigorous analysis of the largest sample of cremated human skeletal remains (348 burial urns, N = 540 individuals) from the Carthaginian Tophet based on tooth formation, enamel histology, cranial and postcranial metrics, and the potential effects of heat-induced bone shrinkage. Most of the sample fell within the period prenatal to 5-to-6 postnatal months, with a significant presence of prenates. Rather than indicating sacrifice as the agent of death, this age distribution is consistent with modern-day data on perinatal mortality, which at Carthage would also have been exacerbated by numerous diseases common in other major cities, such as Rome and Pompeii. Our diverse approaches to analyzing the cremated human remains from Carthage strongly support the conclusion that Tophets were cemeteries for those who died shortly before or after birth, regardless of the cause.

## Introduction

**Figure 1:**
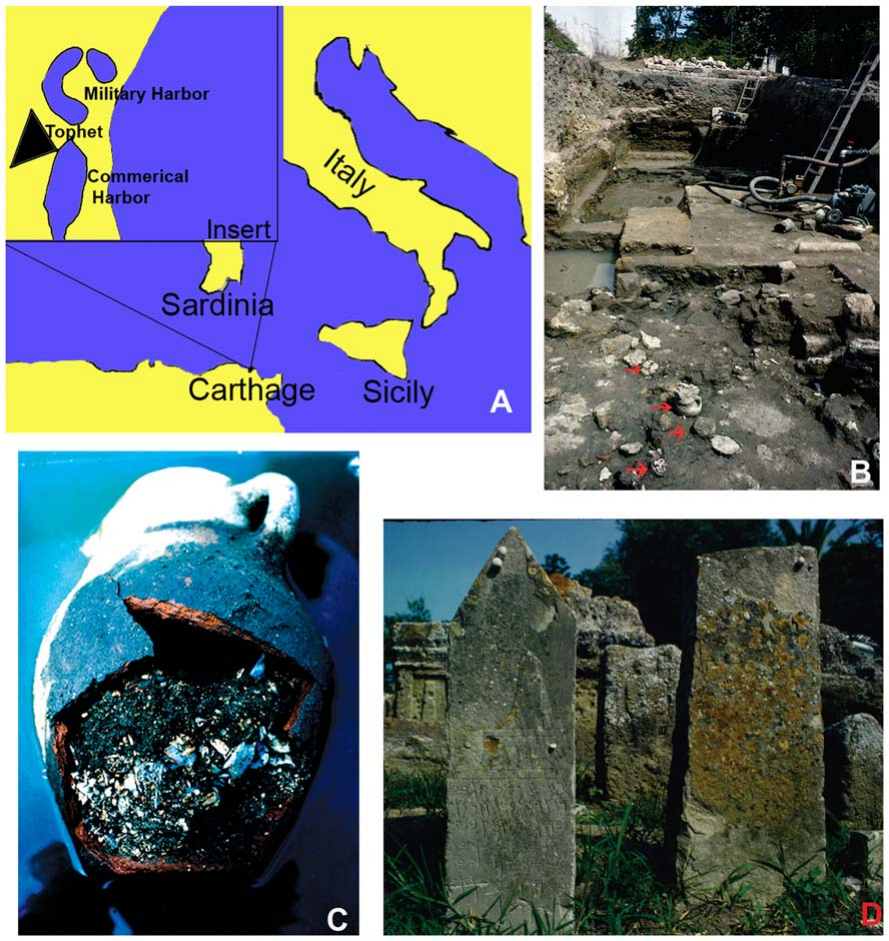
Location of Carthage and excavation of, including objects associated with, the Tophet. A: Map of Western Mediterranean showing location and landmarks of Carthage. B: In order to excavate the Tophet, water had to be continually pumped out of the site (arrows point to urns). C: Broken urn revealing calcined bones and sediment that had seeped in as the water table rose. D: Stelae with different amounts of detail (e.g. one bears an image of an urn and another an inscription).

Some biblical scholars maintain that the Carthaginians frequently and systematically practiced infant sacrifice perhaps as early as Queen Dido's founding of the Phoenician colony on the northern coast of Africa in the 9th or 8th century BCE until 146 BCE, when the Romans won the third and last Punic War [1]–[5]. This interpretation derives from the following: 1) Kleitarchos (3rd c. BCE) described Carthaginians throwing live infants onto a pyre, Diodorus Siculus (1st c. BCE) told of infants' throats being slit prior to cremation, and non-eyewitness reports claim the simultaneous sacrifice and burning of many children; 2) since the Eastern Mediterranean Phoenicians were the Canaanites described in the Old Testament as actually or potentially sacrificing offspring, and specifically first-born males, they continued this ritual as Carthage and its colonies; 3) the centrally situated Carthaginian cemetery contains remains of children and adults while a geographically separate area (the Tophet, Figure 1A) presents small urns (Figure 1B) with burned bones of very young animals (usually lamb or kid), humans (single or multiple individuals) (Figure 1C) and, occasionally, both; 4) inscriptions on some Tophet grave markers (stelae) (Figure 1D) suggest an offering was made to one or both primary deities, Ba'al Hamon and Tanit; and 5) one stela depicts a man, interpreted as a priest, carrying a child. The “all humans were sacrificed” thesis also rests on the argument that, since the animals interred in the Tophet were surely sacrificial victims, so too were the humans also interred in the Tophet [4], [5].

Other biblical scholars [6]–[14], upon reviewing the evidence from the Tophet at Carthage and others at Carthaginian settlements in Cyprus and Sardinia, admit that humans may occasionally have been sacrificed, but also argue that sacrifice alone was not the primary factor underlying human interment in Tophets because: 1) perinatal humans, perhaps stillborn, have tentatively been identified at these sites; 2) the general age-representation of these human samples is consistent with infant mortality, which would have been high; 3) the presence of the very youngest humans in marginally rather than in cross-generational and centrally located cemeteries attests to attributes specific to the young, such as death before at age at which they would have been accepted into society as real individuals; 4) postmortem human cremations were offerings to the deities; and 5) the classical “descriptions” of repeated, large-scale infant sacrifice were exaggerations if not anti-Carthaginian propaganda.

In the latter 1970s, excavations at Carthage were undertaken as part of a UNESCO sponsored, multinational archaeological effort to salvage as much information as possible from the vast site before expansion of building covered everything. The Tunisian Department of Antiquities granted permission to the American Team to excavate and analyze all material–osteological or otherwise–recovered from the Tophet. Once urns were removed from the field, the processing, sorting, osteological analyses of their contents, and the presentation of the results was under the direction of JHS.

Here we provide the results of the first in-depth study not only of the largest sample of the skeletal remains (348 urn contents) from the Tophet at Carthage (summer field seasons 1976 to 1979), but from any Carthaginian Tophet of [see Supporting Information Tables S1, S2]. Our objective was to address the following questions. Were all humans interred in the Tophet sacrificed? Whether sacrificed or merely cremated, how many individuals per event were involved (one, two, or en masse)? Regardless of number of individuals, was each treated with care from pre- to post-cremation? And, as inferred from passages in the Old Testament, were victims exclusively male?

## Methods

Because the water table rose subsequent to use of the Carthaginian Tophet, JHS determined that each excavated urn should be placed in a water-filled bucket until he could extract its contents; otherwise dissolved calcium carbonate would solidify urn contents into a cement-like block as they dried [15], [16]. A weak stream of water aided in removing urn contents onto plastic mesh supported above ground, and in removing adherent silt as urn contents were separated and laid out in a single layer to dry. Bones and teeth, clay that once sealed the urn's mouth, charcoal, urn fragments, and/or amulets or other objects removed from the urn were then sorted [15], [16]. The individualistically stylized and decorated, but poorly fired red-clay urns of the earlier Carthaginian phases were more frequently broken–likely from the weight of water-logged soil and subsequent urn burials–than the more uniform yellow-clay urns of later phases [2], [5], [16].

Since damage to urns and dislodging of the clay seal made possible the loss of material from an urn as well as the intrusion of silts and even bones into the urn [15], soil around the urn was collected to determine the presence of osteological material (JHS). With the exception of the rare small fragment, this “extra-urn” soil was free of burned human bone; on one occasion part of a recent sheep scapula was found inside an urn. The primary intrusive material was, therefore, earth, which seeped in with the water. The complete list of the osteological remains recovered is presented in Tables S1, S2 (Supporting Information). All bones were inspected for evidence of cut marks and other signs of trauma but none was discovered.

**Figure 2:**
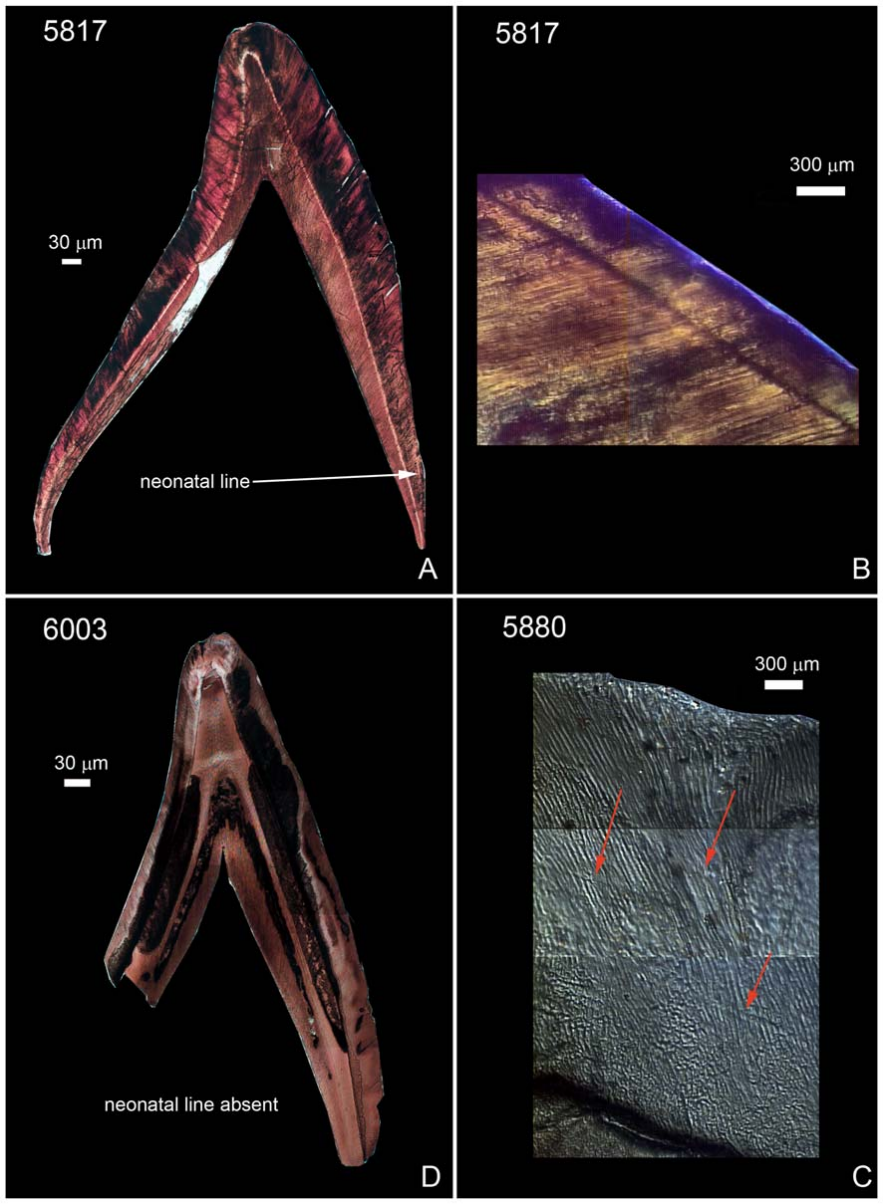
Presence versus absence of neonatal line (NL). A: Longitudinal/buccolingual thin-section of a human upper deciduous central incisor (Urn no. 5817) with a 9.7 µm-thick NL on the buccal (right) side, close to the external enamel margin; the relatively thin postnatal enamel and the distance of the NL from the tooth apex (5.222,5 µm) suggest that the individual survived postpartum at least 10 and perhaps as many as 15 days. B: Close-up of NL (Urn no. 5817). C: Longitudinal/buccolingual thin-section of a human upper deciduous central incisor (Urn no. 6003) lacking an NL. D: Close-up of thin-section of a human upper deciduous central incisor lacking NL (Urn no. 5880; arrows point to Retzius lines). Scale  = 30 µm.

Age estimation was based on comparative measurements of skeletal elements (basilar portion of the occipital or basilaris, sphenoid, petrosal, ischium, and pubis) [17], states of tooth formation [18], and presence or absence of a neonatal line (NL) in the enamel of tooth crowns. The transition from an intra- to extra-uterine environment leaves its mark in deciduous teeth and first permanent molars (the mesial cusp) as an accentuated enamel incremental ring called the neonatal line (NL) [19], [20] (see Figure 2). The NL, which separates the enamel formed during intrauterine life from that formed after leaving the womb, is observable in individuals who survive at least 7 to 10–15 days ex utero [21]–[24].

Given the periodicity of enamel deposition and the fact that prenatal enamel does not normally present accentuated lines, an NL is the first postnatal hypoplasia (i.e. stress-induced alteration of enamel deposition). It thus marks the brief period of disruption of enamel secretion (decrease in daily rates of enamel formation) that occurs immediately postpartum. The emergence of an NL most likely reflects a drop in blood serum calcium values during the first 48 to 72 hours ex-utero [25], [26], as well as the dynamics of a fetus leaving the womb [27].

An NL can be identified easily in ground sections because both the difference in quality between pre- and postnatal enamel and its characteristic location is specific for each tooth class [24], [28]. In incisors, this line extends from the dentino-enamel junction at the cervix (neck) of the crown onto the crown's surface, leaving only a small portion of postnatally formed enamel. In canines and molars, this line is present closer to the incisal/occlusal part of the enamel, with only a small portion of prenatally formed enamel present [29]. Postpartum, the crown thickens via apposition of additional layers of enamel [30].

Analysis of NL presence/absence is routine in forensic investigations, which is noted not only in its increasingly prevalence in analyses of archaeological populations [31]–[34], but especially now in its application to fossil human teeth [35], [36]. Indeed, NL analysis has rapidly become the only currently available osteodontic analytical technique capable of discriminating between infant death during the first postpartum week and the succeeding three weeks.

For this analysis, JHS and FH sent LB and RM well-preserved crowns of deciduous incisors and deciduous molars of 50 individuals, whose estimated ages bracketed the morphologically determined perinatal period and thus the period of transition from in- to ex-utero. Only specimen numbers were provided to LB and RM.

Specimens were cleaned in an ultrasonic bath and embedded in epoxy resin. Longitudinal labio- (bucco-) lingually oriented ground sections were prepared with a diamond blade microtome (Leica 1600) following the protocol of Caropreso et al. [37]. The sectional plane was situated as close as possible to the tip of the dentine horn (for the two deciduous molars, the dentine horns of the mesial cusps). While the quality of the cutting procedure was not always assured because of the condition of the tooth crowns, most specimens were sufficiently preserved enamel to permit reliable NL site-specific assessment.

At least three thin sections per specimen were produced. ∼300 µm-thick slices were subsequently reduced to 80–100 µm with a motorized grinder (Minimet 1000 Buehler), polished, mounted for routine microscopy, and then etched for few seconds with a gel of phosphoric acid in order to enhance enamel microstructure. Of the three slides per tooth, the one with the least diagenetic damage and the most clear-cut microstructure was used in the analysis [33].

Sections were scrutinized under polarized light with an optical transmitted-light microscope (Laborlux S, Leica AG) and images taken with Polaroid Digital Microscope Camera (DMC 1) at 100× and 400×. Contrast enhancement convolution filters (3×3 and 5×5 kernels) produced sharper detail while change in the look-up table function increased site-specific contrasts of intensity profiles. Several partial images (from 7 to 15) were used to reconstruct the entire crown as a digital photomosaic. Because tooth enamel contains significantly less organic material than bone (∼1% vs. ∼20%, respectively), it reacts differently to heat and is less prone to plastic deformation [38]. In addition to its rheological properties, the enamel of unerupted crowns experiences relatively limited cracking and flaking because the structure is buffered against the direct effects of heat by the surrounding bone of the jaw [39]–[41]. While the color of the outermost enamel surface clearly reflects changes in both the burning environment (reduced vs. oxygenic) and temperature [42], the effect of heat on inner enamel microstructure tends to be locally constrained [43], [44]. Within each tooth class, but independent of an individual's sex, the location of the NL is an indirect indicator of gestation length (time of initial mineralization in utero through postpartum), with pre-term birth shifting the line more occlusally [24], [28].

## Results

### Urn Contents

**Figure 3:**
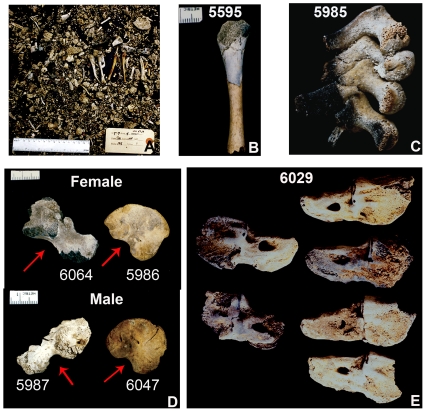
Examples of variably burned bone, female vs male ilia, and duplicate skeletal elements. A: From a single urn, the calcined remains of the remains of a single individual (as reflected in the diversity and non-duplication of preserved skeletal elements). B: Reassociated, partially calcined upper and barely burned middle parts of a right humerus to illustrate the possible degree of fragmentation, dissociation, and consequent disparate crematory fates of parts of the same bone. C: Differentially charred cervical vertebrae still in anatomical position representing one of various indications of incomplete cremation. D: Various pelvic ilia with intact greater sciatic notches (indicated by arrows), whose width (from most to least obtuse) suggests classification as hyperfeminine (upper left), feminine (upper right), hypermasculine (lower left), and masculine (lower right). E: Two left (left) and four right unfused petrosal bones; a straightforward analysis of MNI may suggest the presence of four individuals, but detailed analysis of the urn contents that yielded these petrosals does not provide evidence of four complete individuals in the same urn. (Scales in mm.)

Urns could contain burned bones and teeth of humans, animals (primarily lamb or kid), or both (Table S1). There could be evidence in a single urn of only one human (Figure 3A) or, when number of duplicated parts was used to infer minimum numbers of individuals (MNI) (Figure 3E), as many as seven individuals (Table S2). In cases where one or two individuals were hypothesized present on the basis of MNI, the suite of preserved skeletal elements typically demonstrated that entire individuals had been interred. When, however, MNI indicated the presence of more than two individuals, sufficient numbers of duplicated bones and/or teeth could not be associated on the basis of size or burn pattern to reconstruct with confidence that number of individuals. Thus while multiple duplicates of a skeletal element may indeed reflect the prior existence of that number of individuals, the traditional approach to determining MNI does not provide evidence of an urn containing the complete or nearly complete skeletal remains of each of these individuals. Rather, there was never enough skeletal material to suggest that more than two (relatively) complete skeletons were placed in a single urn, which is inconsistent with a scenario of Carthaginians sacrificing or at least cremating groups of infants whose remains were then carefully collected and interred together in the same urn.

Bones and teeth from the same individual were rarely uniformly charred or calcined, and many were only minimally affected by heat (Figure 3B,C). This irregular burning pattern is consistent with a body on a funeral pyre in which tinder and hot ash were unequal in size and uneven in distribution [45]–to which the presence of burnt small branches in urns attests [16]–and into which bones fell randomly as they separated or burst from the heat and at the same time that pyre-tenders prodded embers to maintain the intensity of the fire [46]. Consequently, when an urn contained nearly complete skeletons, multiple duplicates but little associated skeletal remains, or a single duplicated element amidst the relatively complete remains of one or two perinates, we could infer with confidence that if individuals had been dealt with separately, such attention did not persist beyond cremation. Instead, we suggest, bones and teeth that fell deep into the pyre were left behind and inadvertently collected with the remains of subsequently cremated individuals. Similarly, if multiple cremations had occurred, either simultaneously or in short succession, there was obviously no attempt to prevent comingling of bones and teeth from different individuals.

### Determination of Sex

Seventy pelvic ilia were sufficiently preserved for visual assessment of sex, for which we relied on angle and depth of the greater sciatic notch and, when preserved sufficiently to be scrutinized, curvature of the iliac crest (Figure 3D). In Schutkowski's [47] study of a sample of children sexes and ages-at-death were well-documented, greater sciatic notch angle correctly assigned, respectively, males 95% and females 71.4%, notch depth 81.2% and 76.5%, and crest curvature 81.2% and 62.1% of the time. In our sample of ilia, 26 very probably and one questionably represented male, and 38 probably and two more questionably female (Table 1); three specimens were indeterminate. Given the likelihood that at least some individuals we identified as female were indeed female, the hypothesis of first-born males being the focus of a Carthaginian ritual of sacrifice is falsified.

**Table 1:** Probable Sex of Human Remains [based on Greater Sciatic Notch (GSN) width], Carthaginian Tophet.

Urn	Individual	Side	GSN width (in Degrees)	GSN Morphology	Suggested Sex
*17	1	R		Narrow and Deep	Male
*20	1	L	113	Narrow and Deep	Male
*36	1	L	100	Wide and Shallow	Female
*37	1	R	98	Wide and Shallow	Female
*172	1	L	93	Wide and Deep	Female
*180	1	R		Deep (?)	Male (?)
*187	1	R		Wide and Shallow	Female
*213	1	L		Narrow and Deep	Male
*222	1	R	95	Narrow and Deep	Male
*232	1	R	98	Wide and Shallow	Male
5409	1	R	98	Narrow and Shallow	Female
5414	1	R		Wide and Shallow	Female
5529	1	L		Wide and Shallow	Female
5538	1	R	98	Narrow and Deep	Male
5545	2	L		Wide and Shallow	Female
5545	1	L		Wide and Shallow	Female
5577	1	R		Wide and Shallow	Female
5579	1	L	101	Wide and Shallow	Female
5623	1	R	100	Narrow and Deep	Male
5817	1	R		Narrow and Deep	Male
5818	1	L	101	Deep (?)	Indeterminate
5824	1	R	90	Narrow and Deep	Male
5827	1	R	107	Wide and Shallow	Female
5829	1	L	109	Narrow and Deep	Male
5830	1	R	107	Deep (?)	Indeterminate
5835	1	L	103	Wide and Shallow	Female
5841	1	R		Wide and Shallow	Female
5843	1	R	116	Wide and Shallow	Female
5849	1	R	110	Wide and Shallow	Female
5850	1	L	72	Narrow and Deep	Male
5881	1	L		Wide and Deep	Male
5887	2	R	119	Shallow	Male
5887	1	R	114	Narrow and Deep	Male
5890	1	L	93	Wide and Deep	Male
5893	1	R		Narrow and Deep	Male
5894	1	L		Narrow and Deep	Male
5895	1	R	103	Wide and Shallow	Female
5903	1	R		Wide and Deep	Female
5920	1	L		Wide and Shallow	Female
5932	1	R		Wide and Shallow	Female
5945	1	L	114	Wide and Shallow	Female
5946	1	R		Shallow	Female
5948	1	L	102	Wide and Shallow	Female
5959	1	R	95	Wide and Shallow	Female
5962	1	L	116	Wide and Shallow	Female
5963	1	R	103	Narrow and Deep	Indeterminate
5967	1	R	127	Wide and Shallow	Female
5971	1	R	100	Wide and Shallow	Female
5984	1	L		Narrow and Deep	Male
5986	1	R	104	Wide and Shallow	Female
5987	1	R	110	Wide and Deep	Male
5991	1	L	123	Wide and Shallow	Female?
5992	1	R	129	Wide and Shallow	Female
5995	1	R		Narrow and Deep	Female
6000	1	R	110	Wide and Shallow	Female
6000	1	R		Wide and Shallow	Female
6001	1	L		Wide and Shallow	Female?
6028	1	R	105	Wide and Shallow	Female
6029	1	R		Wide and Shallow	Female
6032	1	L		Narrow and Deep	Male
6037	1	L	105	Wide and Deep	Female
6043	1	L	96	Narrow and Deep	Male
6047	1	R	97	Narrow and Deep	Male
6064	1	R	135	Wide and Shallow	Female
6068	1	L		Narrow and Deep	Male
6379	1	R	103	Wide and Shallow	Female
6380	1	R		Narrow and Deep	Male
6392	1	L	95	Narrow and Deep	Male
6396	1	L		Wide and Shallow	Female
6398	1	L	101	Narrow and Deep	Male

Key: *  =  Basket Number; R  =  Right; L: Left.

### Estimation of Age: Tooth Formation and Osteometrics

Only bones and teeth and tooth crowns that were preserved sufficiently intact to provide an accurate (not estimated) measurement were used in our estimation of age. Based on skeletal measurements (of the basilar portion of the occipital or basilaris, sphenoid, petrosal, ischium, and pubis; Tables S3, S4) [17], as well as relative states of tooth formation (Table S2) [18], most of the sample fell within the range of 2 to 12 postnatal months, clustering between 2 and 5 months at death (Table S2). At least another 20% of the sample (depending on the representation of the specific skeletal element) could be identified as prenatal. These results are consistent with modern infant mortality data [48], [49]. We ruled out misclassifying infants of “low birth weight” (LBW) as prenatal because, while mortality is 40% higher in perinates <2500 gm than infants of normal birth weight [50], LBW is not reflected in diminished bone length or retarded tooth development [51].

Although experiments on heat-induced bone shrinkage were not done in the manner of Carthaginian cremation, we nonetheless thought it prudent to consider them. Most of these studies used ovens rather than fire as well as dry and defleshed green rather than fleshed bone [e.g. 52]–[54]. In all cases, bone shrinkage was minimal. Richard [55] did, however, cremate parts of human infant cadavers, but focused only on temperature and degree of bone carbonation and calcination. Baby [56], who cremated fleshed adult human remains, concluded that bone size was either not, or at most only minimally, altered. Buikstra and Swegle [57] cremated fleshed adult animal remains and found that while bone shrinkage could be as much as 6%, in general, bone size was minimally affected. Dokládal [58] compared bones from cremated halves of five adult cadavers with their uncremated counterparts and reported shrinkage between 5 and 12%. Muller's [59] cremations of defleshed human fetal and newborn bones suggest shrinkage could reach 10%.

**Figure 4:**
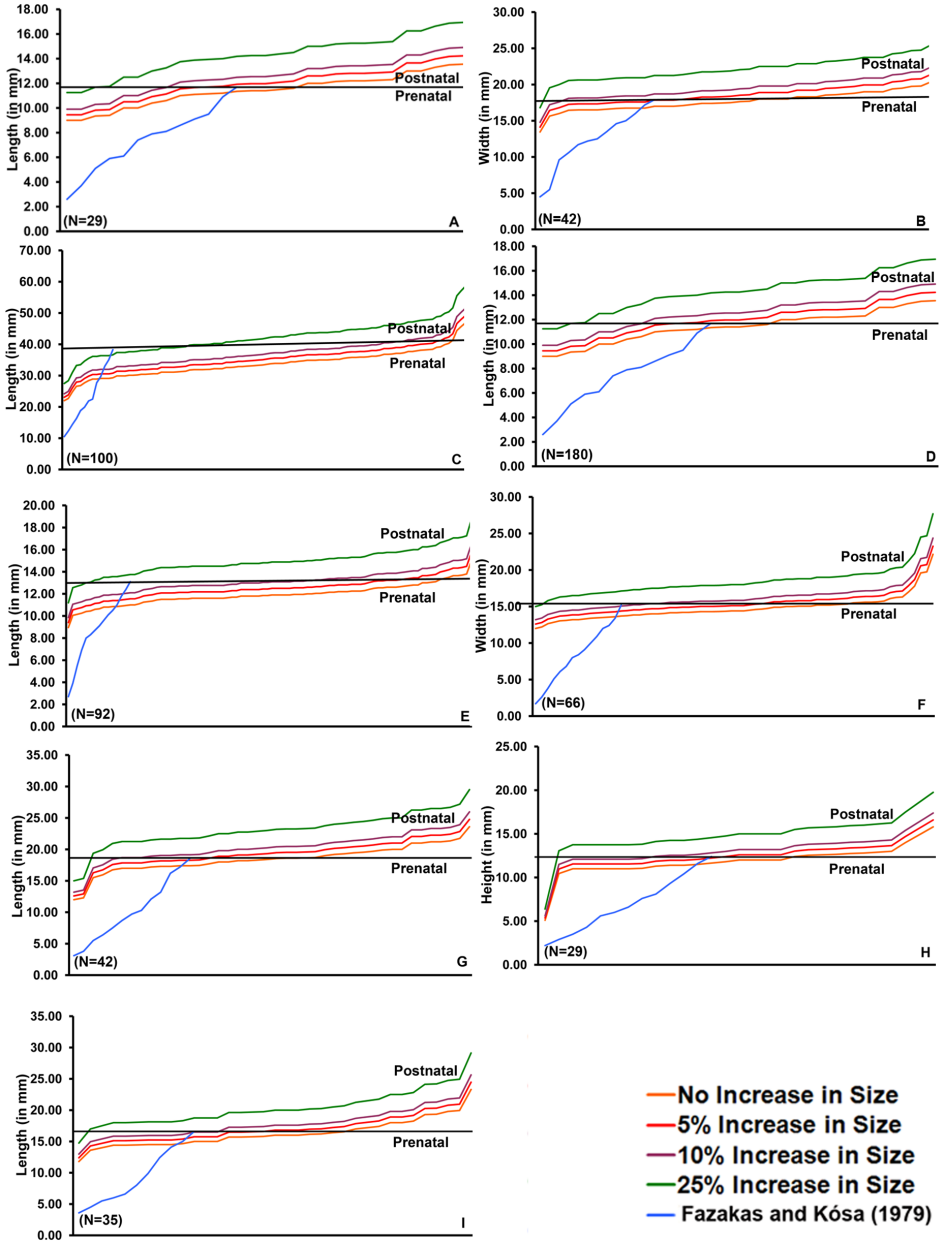
Plots of ages-at-death determined by actual (maximum) and incrementally increased size of skeletal elements sufficiently preserved for accurate measurement. A: Hypophyseal fossa length. B: Hypophyseal fossa width. C: Petrosal length. D: Petrosal width. E: Pars basilaris length. F: Pars basilaris width. G: Ischium length. H: Ischium height. I: Pubis length. In the graph, the same bones are compared to data from Fazekas and Kósa [17] and also increased by 5, 10 and 25%. The horizontal line in each represents birth.

Although some Carthaginian perinates' bones were barely charred–and thus their exposure to heat minimal [46]–we increased all of our measurements by 5, 10 and then an extreme 25% in order to account for any possible shrinkage (Figure 4). Even at 25% increase in size, most of our analyses still classified some individuals as prenates and thus not available for sacrifice.

### Estimation of Age: Neonatal Line (NL) Analysis

In the Carthaginian sample, NL thickness ranged from 6.3 to 14.5 µm, with a mean of 10.1 µm (±2.76 µm). Comparative estimates obtained by the same investigative methods on deciduous teeth of all morphological classes were available from 124 crowns representing 102 modern European children [43], [60] and from 209 crowns representing 109 children (aged 6 months to 9 years) buried at the Imperial Roman cemetery of Isola Sacra [31], [60]. In the modern sample, NL thickness ranged from 6.5 to 50.9 µm and the mean value corresponded to 17.3 µm (±7.97 µm). In the archaeological sample, the range of variation range 9–36 µm with a mean of 16.7 µm (±4.40 µm). Additional values from a modern sample of 147 children ranged from 10 to 24 µm [27].

**Figure 5:**
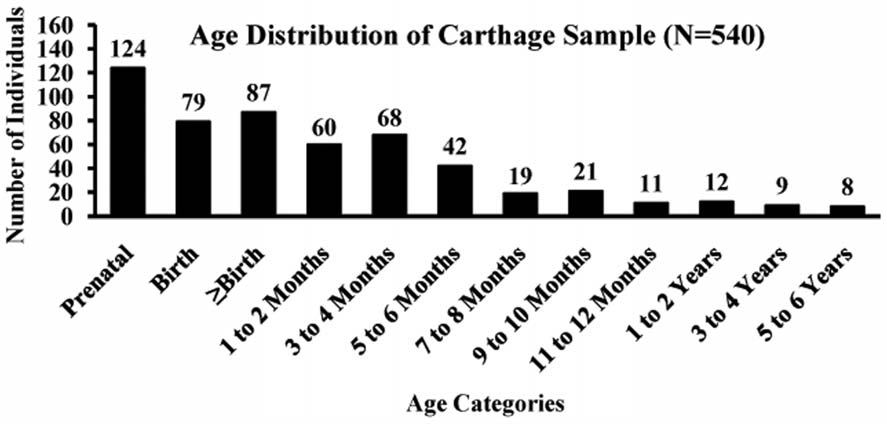
Distribution of Ages-at-Death Based on Analysis of Human Remains, Carthaginian Tophet.

An NL results from perturbation in matrix deposition of enamel prisms reflecting stress in the transition from an intra- to extra-uterine environment (Figure 2), which does not always correspond to parturition following a full-term pregnancy [61]. Given the periodicity of enamel deposition, a newborn must survive at least 7 and even as many as 10 to 15 extra-uterine days in order for an NL to emerge fully. A definitive NL was observed in 24 Carthaginian specimens (Table 2); the amount of subsequent enamel deposition suggests these individuals survived at least 2 weeks postpartum. An NL was absent in 26 Carthaginian specimens (Table 2), which suggests that these individuals were either stillborn, spontaneously aborted, or died during the first extra-uterine week. Unambiguous counts and measurements of daily enamel cross-striations, which provide information on the timing and rate of enamel deposition and thus indirect evidence of gestation length [31], [33], could not be obtained on this sample. However, because other analyses in our study indicate the presence of individuals who had not reached full term, we suggest that individuals lacking an NL probably fall into the prenatal category because comparison of morphological/metric and NL age estimates demonstrates that when they differed, the histological (NL) age more frequently over-aged individuals than did morphological age (M<H 22%, M>H 10%; see Table 3). Consequently, if we include with the prenates those individuals who did not survive beyond one or even two weeks postpartum, we must conclude that a significant number of individuals could not have been sacrificed because they were either not alive or not yet old enough to be considered viable sacrificial entities [7], [8], [10], [13] (Figure 5).

**Table 2:** Neonatal Line (NL) Analysis of Human Deciduous Teeth (N = 50), Carthaginian Tophet.

Urn	Tooth	NL	NL thickness (µm)^a^	Notes
*198	upper central incisor	Present	-	NL observed lingually
*200	upper central incisor	Present	9.6	NL observed labially, close to outer margin
*208	upper central incisor	Present	6.7	almost complete NL, observed both labially and lingually
*223	lower second molar	Absent		
2522	incisor (indeterminate)	Present	-	NL observed lingually, close to outer margin
5163	upper lateral incisor	Absent		
5331	incisor (indeterminate)	Absent		
5410	lower central incisor	Absent		
5531	upper lateral incisor	Present		NL observed lingually, close to the outer margin
5570	lower central incisor	Present		NL observed labially, close to cervix
5576	upper central incisor	Absent		
5582	lower central incisor	Absent		
5583	incisor (indeterminate)	Present	12.1	NL observed lingually, close to cervix
5587	upper central incisor	uncertain		a possible NL-like accentuated ring close to the labial cervix
5591	incisor (indeterminate)	Present	10.8	NL observed labially, close to the cervix
5599	upper central incisor	Present	14.3	NL observed labially
5625	upper central incisor	Present	-	NL observed lingually, close to the outer margin
5817	upper central incisor	Present	9.7	NL observed labially, close to external margin
5818	incisor (indeterminate)	Absent		
5829	upper central incisor	Present	8.2	NL observed labially, close to cervix
5831	upper lateral incisor	Absent		
5834	upper lateral incisor	Absent		
5840	upper central incisor	Absent		
5852	upper central incisor (?)	Present	14.5	NL observed both labially and lingually
5855	lower central incisor	Absent		
5862	upper first molar	Present		NL observed buccally and occlusally
5868	upper lateral incisor	Present	8.2	NL observed both labially and lingually
5880	lower central incisor	Absent		
5883	upper central incisor	Present		NL observed labially, close to the outer margin
5902	upper central incisor	Absent		
5948	upper lateral incisor	Present		NL observed lingually, close to the outer margin
5952	upper lateral incisor	Absent		
5966	upper central incisor (?)	Absent (?)		crown fragment
5971	incisor (indeterminate)	Absent		
5991	upper central incisor	Present		NL observed labially
5998	upper central incisor	Absent		
6003	upper lateral incisor (?)	Absent (?)		crown fragment
6023	upper lateral incisor	Present		NL observed lingually
6039	lower lateral incisor	Absent		
6049	upper lateral incisor	Absent		
6051	upper lateral incisor	Absent		
6054	upper central incisor	Absent		
6055	upper lateral incisor	Present	6.3	NL observed labially, close to outer margin
6058	incisor (indeterminate)	Present		NL observed labially, close to external margin
6068	upper lateral incisor	Absent		
6069	incisor (indeterminate)	Absent		
6070	upper central incisor	Present		NL observed lingually, ≈ at mid-crown
6393	upper lateral incisor	Absent		
6398	upper lateral incisor	Absent		
6399	lower lateral incisor	Present	11.2	NL observed labially

Key: *  =  Basket Number; ^a^: mean value.

**Table 3:** Comparison of Ages-at-Death Determined by Neonatal Line [Histological (H)] and Morphological (M) Analyses of Human Deciduous Teeth, Carthaginian Tophet.

Urn	Neonatal Line	Morphology	H versus M
3178	Absent	≤Birth	M = H
5163	Absent	≤Birth	M = H
5331	Absent	?	Histological Age Only
5410	Absent	Birth	M>H
5576	Absent	Late Third Trimester	M = H
5582	Absent	≤Birth	M = H
5818	Absent	Late Third Trimester	M = H
5831	Absent	Late Third Trimester	M = H
5834	Absent	≤Birth	M = H
5840	Absent	Late Third Trimester	M = H
5855	Absent	Late Third Trimester	M = H
5880	Absent	Late Third Trimester	M = H
5902	Absent	≤Birth	M = H
5952	Absent	Late Third Trimester	M = H
5966	Absent	∼Birth	M>H
5971	Absent	Late Third Trimester	M = H
5998	Absent	≤Birth	M = H
6003	Absent	Late Third Trimester	M = H
6039	Absent	Late Third Trimester	M = H
6049	Absent	Birth to 1 Month	M>H
6051	Absent	∼Birth	M>H
6054	Absent	≤Birth	M = H
6068	Absent	∼Birth	M = H
6069	Absent	Birth	M>H
6393	Absent	∼Birth	M = H
6398	Absent	≤Birth	M = H
3159	Present	1 to 2 months postnatal	M = H
3167	Present	∼Birth	M = H
3163	Present	Late Third Trimester	M<H
2522	Present	?	Histological Age Only
5531	Present	∼Birth	M = H
5570	Present	∼Birth	M = H
5583	Present	?	Histological Age Only
5587	Present	Late Third Trimester	M<H
5591	Present	?	Histological Age Only
5599	Present	∼Birth	M = H
5625	Present	2 Months	M = H
5817	Present	?	Histological Age Only
5829	Present	<Birth	M<H
5852	Present	Birth	M = H
5862	Present	≤Birth	M<H
5868	Present	≤Birth	M<H
5883	Present	Late Third Trimester	M<H
5948	Present	Birth	M = H
5991	Present	≤Birth	M<H
6023	Present	≤Birth	M<H
6055	Present	Late Third Trimester	M<H
6058	Present	≤Birth	M<H
6070	Present	∼Birth	M = H
6399	Present	≤Birth	M<H

Key: M = H: Morphological and Histological ages similar; M>H: Morphological age advanced compared to Histological age; M<H: Histological age advanced compared to Morphological age.

## Discussion

The identification of prenatal individuals in the Carthaginian Tophet sample is consistent with current data from modern-day studies on the incidence of stillbirth and spontaneous abortion as being the primary contributors to “reproductive wastage” [62], as well as with recent data on infant mortality [48], [49]. For example, in England and Wales from 1969 to 1976, 48.4% of 6517 deaths within two weeks of live birth occurred between 30 minutes and 24 hours and 39.3% between 7 and 13 days [61]. These statistics easily accommodate our results.

Infectious diseases known to lead to stillbirth include smallpox, vaccinia, and listeriosis; those resulting in prematurity and perinatal mortality include severe viral infections and malaria [49]. Noninfectious diseases resulting in stillbirth, abortion, or preterm delivery include cholestasis, hypertension, toxemia, and renal disease [50]. The Carthaginians were probably exposed to and susceptible to all of these afflictions. If conditions of sanitation at Carthage, including management of water supply and human and animal excreta, were similar to those at Pompeii, Ostia, and Rome [63], the Carthaginians would also have been potential victims to and vectors of cholera, dysentery, gastroenteritis, infectious hepatitis, leptospirosis, typhoid, and parasitic intestinal infestations, most of which result in severe dehydration, which is a common cause of infant death [50].

In sum, while the Carthaginians may occasionally have practiced human sacrifice, as did other circum-Mediterranean societies [1], [63], [64], our analyses do not support the contention that all humans interred in the Tophet had been sacrificed. Rather, it would appear that the Carthaginian Tophet, and by extension Tophets at Carthaginian settlements in general, were cemeteries for the remains of human prenates and infants who died from a variety of causes and then cremated and whose remains, sometimes on a catch-as-catch-can basis, interred in urns. Following widespread practice at this time in history, it is likely that at least some, if not all, of the cremated animal remains represent sacrificial offerings.

## Supporting Information

Table S1 Species Identification of Skeletal Remains from Urns, Carthaginian Tophet.
(0.33 MB DOC)

Table S2 Demographic Profile of Human Remains, Carthaginian Tophet.
(0.93 MB DOC)

Table S3 Dimensions of Human Cranial Bones (in mm.), Carthaginian Tophet.
(0.33 MB DOC)

Table S4 Dimensions of Human Pelvic Bones (in mm.), Carthaginian Tophet.
(0.12 MB DOC)

## References

[1] Brown S (1991). Late Carthaginian Child Sacrifice and Sacrificial Monuments in their Mediterranean Context..

[2] Harden D (1963). The Phoenicians..

[3] Mosca PG (1975). Child Sacrifice in Canaanite and Israelite Religion..

[4] Stager LE, Greene JA (2007). Child sacrifice: yes, children of Phoenician/Punic Carthage were sacrificed to the gods.. http://phoeniciaorg/childsacrificehtml.

[5] Stager LE, Wolf SR (1984). Child sacrifice at Carthage: Religious rite or population control.. Biblical Archaeology Review.

[6] Agelarakis AP, Kanta A, Stampolidis N, Karageorghis V, Stampolidis N (1998). The osseous record in the Western Necropolis of Amathous: an archaeo-anthropological investigation.. Eastern Mediterranean Cyprus-Dodecanses-Crete 16th-6th cent BC.

[7] Benichou-Safar H (1981). A propos des ossements humains du *tophet* de Carthage.. Rivista di Studi Fenici.

[8] Bartoloni P (2006). Il Tophet: un pietoso rito offuscato da troppo miti.. Darwin Quaderni.

[9] Conte S (2007). Child sacrifice: children of Phoenician Punic Carthage were not sacrificed to the gods.. http://phoeniciaorg/childsacrificehtml.

[10] Fantar MH (2007). Child sacrifice: child of Pheonician Punic Carthage were not sacrificed.. http://phoeniciaorg/childsacrificehtml.

[11] Fedele F, Foster C (1988). Tharros ovicaprini sacrificiali e rituale del Tofet.. Rivista di Studi Fenici.

[12] Lancel S (1995). Carthage: a history..

[13] Moscati S (1987). Il sacrificio punico dei fanciulli: realta or invenzione.. Quaderni dell'Accademia Nazionale dei Lincei.

[14] Simonetti A (1983). Sacrifici umani e uccisioni rituali nel mondo Fenicio-Punico: il contributo delle fonti letterarie.. Rivista di Studi Fenici.

[15] Schwartz JH (1993). What the Bones Tell Us..

[16] Schwartz JH (1989). The Tophet and “sacrifice” at Phoenician Carthage: an osteologist's perspective.. Terra.

[17] Fazekas IG, Kósa F (1979). Forensic Fetal Osteology..

[18] Schwartz JH (2007). Skeleton Keys: an introduction to human skeletal morphology, development, and analysis..

[19] Rushton A (1939). The birifrangence of deciduous tooth enamel formed before and after birth.. Britannic Dental Journal.

[20] Kronfeld R, Schour I (1939). Neonatal dental hypoplasia.. Journal of the American Dental Association.

[21] Weber DF, Eisenmann D (1971). Microscopy of the neonatal line in developing human enamel.. American Journal of Anatomy.

[22] Whittaker DK, Richards D (1978). Scanning electron microscopy of the neonatal line in human enamel.. Archives of Oral Biology.

[23] Levine RS, Turner EP, Dobbing J (1979). Deciduous teeth contain histories of developmental disturbances.. Early Human Development.

[24] Skinner MF (1992). Gestation length and location of the neonatal line in human enamel.. Journal of Paleopathology.

[25] Seow WK (1986). Oral implication of premature birth.. Australian Dental Journal.

[26] Ranggard L, Norén JG, Nelson N (1994). Clinical and histologic appearance in enamel of primary teeth in relation to neonatal blood ionized calcium values.. Scandinavian Journal of Dental Research.

[27] Eli I, Sarnat H, Talmi E (1989). Effect of the brith process on the neonatal line in primary tooth enamel.. Pediatric Dentistry.

[28] Skinner MF, Dupras T (1993). Variation in birth timing and location of the neonatal line in human enamel.. Journal of Forensice Sciences.

[29] Teivens A, Mornstad H, Norén JG, Gidlund E (1996). Variation in birth timing and location of the neonatal line in human enamel.. Forensic Science International.

[30] Shellis RP (1984). Variations in growth of the enamel crown in human teeth and a possible relationship between growth and enamel structure.. Archives of Oral Biology.

[31] Rossi PF, Bondioli L, Geusa G, Macchiarelli R (1999). Osteodental Biology of the People of Portus Romae (Necropolis of Isola Sacra, 2nd–3rd Cent. AD). I. Enamel Microstructure and Developmental Defects of the Primary Dentition..

[32] Smith P, Avishai G (2005). The use of dental criteria for estimating postnatal survival in skeletal remains of infants.. Journal of Archaeological Science.

[33] FitzGerald C, Saunders S, Bondioli L, Macchiarelli R (2006). Health of infants in an Imperial Roman skeletal sample: perspective from dental microstructure.. American Journal of Physical Anthropology.

[34] Macchiarelli R, Bondioli L, Caropreso S, Mazurier A, Merceron G (2006). The oldest human remains from the Beagle Channel, Tierra del Fuego.. International Journal of Osteoarchaeology.

[35] Macchiarelli R, Bondioli L, Debénath A, Mazurier A, Merceron G (2006). How Neanderthal teeth grew.. Nature.

[36] Smith TM, Toussaint M, Reid DJ, Olejniczak AJ, Hublin J-J (2007). Rapid dental development in a Middle Paleolithic Begian Neanderthal.. Proceedings of the National Academy of Sciences (USA).

[37] Caropreso S, Bondioli L, Capannolo D, Cerroni L, Macchiarelli R (2000). Thin sections for hard tissues biology: a new procedure.. Journal of Microscopy.

[38] Gejvall N-G (1969). Cremations. In: Brothwell D, Higgs E, Clark G, editors. Science in Archaeology. 2nd ed..

[39] McKinley JI (1994). The Anglo-Saxon Cemetery at Spong Hill, North Elmham. Part VIII: the Cremations..

[40] Hillson S (1996). Dental Anthropology..

[41] Gatto E (2003). La Place de la Crémation dans le Traitement des Défunts à la Fin du Néolithique en France. Outils Méthodologiques et Etudes de Sites.. Bordeaux: Université de Bordeaux.

[42] Susini A (2003). Etude des Caractéristiques Biophysiques des Tissus Calcifiés Humains Soumis à des Traitements Thermiques: Applications Anthropologiques et Médicales..

[43] Macchiarelli R, Petrone PP, Bondioli L (1996). I resti ossei combusti. Analisi morfologica ed istomorfometrica.. Atti e Memorie della Società Magna Grecia.

[44] Bondioli L, Macchiarelli R, Fedele F, Petrone PP (1999). Indagini istomorfometriche sullo scheletro e sui denti di San Paolo Belsito.. Un'Eruzione Vesuviana 4000 Anni Fa.

[45] McKinley JI, Roberts CA, Lee F, Bintliff J (1989). Cremations: expectations, methodologies and realities.. Burial Archaeology: Current Research, Methods and Developments.

[46] Gejvall N-G, Brothwell D, Higgs E (1963). Cremations.. Science in Archaeology: A Comprehensive Survey of Progress and Research.

[47] Schutkowski H (1993). Sex determination of infant and juvenile skeletons I. Morphognostic features.. American Journal of Physical Anthropology.

[48] Saunders SR, Barrans L, Hoppa RD, Firzgerald CM (1999). What can be done about the infant category in skeletal sample?.

[49] Taylor CM, Pernoll ML, Pernoll ML, Benson RC (1987). Normal pregnancy & prenatal care.. Current Obstetric & Gynecologic Diagnosis & Treatment 1987.

[50] Behrman RE, Shiono PH, Fanaroff AA, Martin RJ (1997). Neonatal risk factors.. Neonatal-Perinatal Medicine: Diseases of the Fetus and Infant. St.

[51] Jaya DS, Kumar NS, Bai LS (1995). Anthropometric indices, cord length and placental weight in newborns.. Indian Pediatrics.

[52] Pointek J (1976). The process of cremation and its influence on the morphology of bones in light of results of experimental research.. Achaeologia Polski.

[53] Shipman P, Forster G, Schoeninger M (1984). Burnt bones and teeth: an experimental study of colour, morphology, crystal structure and shrinkage.. Journal of Archaeological Science.

[54] Thurman MD, Willmore LJ (1981). A replicative cremation experiment.. North American Archaeologist.

[55] Richard J (1961). Etude Médico-Légale des Urnes Sacrificielles Puniques et de leur Contenu..

[56] Baby RS (1954). Hopewell Cremation Practices..

[57] Buikstra JE, Swegle M, Bonnischen R, Sorg MH (1989). Bone modification due to burning: experimental evidence.. Bone Modification.

[58] Dokládal M, Novotny N (1971). A further contribution to the morphology of burned bones.. Proceedings of the Anthropological Congress Dedicated to Ales Hrdlicka.

[59] Muller M (1938). La calcinations du foetus en medicine légale..

[60] Bondioli L, Macchiarelli R (1999). Neonatal line thickness and delivery at Isola Sacra (2nd–3rd cent. AD, Rome, Italy).. American Journal of Physical.

[61] Chalmers J, Macfarlane A, Wharton BA (1980). Interpretation of perinatal statistics.. Topics in Perinatal Medicine.

[62] Durfee RB, Wharton BA (1987). Obstetric complications of pregnancy.. Topics in Perinatal Medicine.

[63] Scobie A (1986). Slums, sanitation, and mortality in the Roman world.. Klio.

[64] Moscati S (1965). The World of the Phoenicians..

