# Supplementary material for: Skeletal Remains from Punic Carthage Do Not Support Systematic Sacrifice of Infants
Source: PLoS One. 2010 Feb 17;5(2):e9177. doi: 10.1371/journal.pone.0009177 (PMC2822869; doi:10.1371/journal.pone.0009177)
Supplement: Table S1 — Species Identification of Skeletal Remains from Urns, Carthaginian Tophet. (0.33 MB DOC) [file pone.0009177.s001.doc]

| **Urn #** | ***Homo sapiens*** | ***Ovis***  **(lamb)** | ***Capra***  **(kid)** | | ***Ovis/Capra***  **(indeterminate)** | | **Faunal**  **(other)** |
| --- | --- | --- | --- | --- | --- | --- | --- |
|  |  |  |  | |  | |  |
| *158 | X | - | - | | - | | - |
| *192 | X | - | - | | - | | - |
| *198 | X | - | - | | - | | - |
| *200 | X | - | - | | - | | - |
| 2390 | X | - | - | | - | | - |
| 2844 | X | - | - | | - | | - |
| 2845 | X | - | - | | - | | - |
| 2846 | X | - | - | | - | | - |
| 2848 | X | - | - | | - | | - |
| 3091 | X | - | - | | - | | - |
| 3092 | X | - | - | | - | | - |
| 3093 | X | - | - | | - | | - |
| 3159 | X | - | - | | - | | - |
| 3160 | X | - | - | | - | | - |
| 3163 | X | - | - | | - | | - |
| 3164 | X | - | - | | - | | - |
| 3165 | X | - | - | | - | | - |
| 3167 | X | - | - | | - | | - |
| 3168 | X | - | - | | - | | - |
| 3169 | X | - | - | | - | | - |
| 3171 | X | - | - | | - | | - |
| 3176 | X | - | - | | - | | - |
| 3177 | X | - | - | | - | | - |
| 3178 | X | - | - | | - | | - |
| 3179 | X | - | - | | - | | - |
| 3180 | X | - | - | | - | | - |
| 3185 | X | - | - | | - | | - |
| 3186 | X | - | - | | - | | - |
| 3187 | X | - | - | | - | | - |
| 3188 | X | - | - | | - | | - |
| 3190 | X | - | - | | - | | - |
| 3191 | X | - | - | | - | | - |
| 3192 | X | - | - | | - | | - |
| 4434 | X | - | - | | - | | - |
| 4435 | X | - | - | | - | | - |
| 4436 | X | - | - | | - | | - |
| 4438 | X | - | - | | - | | - |
| 4612 | X | - | - | | - | | - |
| 4613 | X | X | - | | - | | - |
| 4614 | X | - | - | | - | | - |
| 4615 | X | - | X | | - | | - |
| 4937 | X | X | - | | - | | X |
| 4938 | X | X | - | | - | | X |
| 4939 | X | - | - | | - | | - |
| 4940 | X | - | - | | - | | - |
| 4941 | X | - | - | | - | | - |
| 4942 | X | - | - | | X | | - |
| 4957 | X | - | - | | - | | X |
| 4958 | X | - | - | | - | | - |
| 5171 | X | - | - | | - | | - |
| 5172 | X | - | | - | | - | - |
| 5173 | X | - | | - | | - | - |
| 5174 | X | - | | - | | - | - |
| 5189 | X | - | | - | | - | - |
| 5190 | X | - | | - | | - | - |
| 5191 | X | - | | - | | - | - |
| 5192 | X | - | | - | | - | - |
| 5407 | X | - | | - | | - | - |
| 5408 | X | X | | - | | - | - |
| 5409 | X | - | | - | | - | - |
| 5410 | X | - | | - | | - | - |
| 5412 | X | - | | - | | - | - |
| 5414 | X | - | | X | | - | - |
| 5415 | X | - | | - | | - | - |
| 5416 | X | - | | - | | - | - |
| 5417 | X | - | | - | | - | - |
| 5419 | X | - | | - | | - | - |
| 5516 | X | - | | X | | - | - |
| 5517 | X | - | | - | | - | - |
| 5519 | X | - | | - | | - | - |
| 5521 | X | - | | - | | - | - |
| 5522 | X | - | | - | | - | - |
| 5524 | X | - | | - | | - | - |
| 5525 | X | - | | - | | - | - |
| 5526 | X | - | | - | | - | - |
| 5527 | X | X | | - | | - | - |
| 5528 | X | - | | - | | - | - |
| 5529 | X | - | | - | | - | - |
| 5531 | X | - | | - | | - | - |
| 5532 | X | - | | - | | - | - |
| 5533 | X | - | | - | | - | - |
| 5538 | X | - | | - | | - | - |
| 5541 | X | - | | - | | - | - |
| 5543 | X | - | | - | | - | - |
| 5547 | X | - | | - | | X | - |
| 5548 | X | - | | - | | - | - |
| 5551 | X | - | | - | | - | - |
| 5553 | X | - | | - | | - | - |
| 5558 | X | - | | - | | - | - |
| 5559 | X | - | | - | | - | - |
| 5561 | X | - | | - | | - | - |
| 5563 | X | - | | - | | - | - |
| 5565 | X | - | | - | | - | - |
| 5566 | X | - | | - | | - | - |
| 5567 | X | - | | X | | X | - |
| 5568 | X | - | | - | | - | X |
| 5569 | X | - | | - | | - | - |
| 5570 | X | - | | - | | - | - |
| 5571 | X | - | | - | | - | - |
| 5573 | X | - | | - | | - | - |
| 5574 | X | - | | - | | - | X |
| 5575 | X | - | | - | | - | - |
| 5576 | X | - | | - | | X | - |
| 5577 | X | - | | - | | - | - |
| 5578 | X | - | | - | | - | - |
| 5579 | X | - | | - | | X | - |
| 5580 | X | - | | - | | - | - |
| 5581 | X | - | | - | | - | - |
| 5582 | X | - | | - | | X | - |
| 5584 | X | - | | - | | - | - |
| 5586 | X | - | | - | | - | - |
| 5587 | X | - | | - | | - | - |
| 5588 | X | - | | - | | - | - |
| 5589 | X | - | | - | | - | - |
| 5590 | X | - | | - | | - | - |
| 5593 | X | X | | - | | - | - |
| 5594 | X | - | | - | | X | - |
| 5595 | X | - | | - | | - | - |
| 5596 | X | - | | - | | - | - |
| 5597 | X | - | | - | | X | - |
| 5599 | X | - | | - | | - | - |
| 5600 | X | - | | - | | - | - |
| 5602 | X | - | | - | | - | - |
| 5603 | X | - | | - | | - | - |
| 5604 | X | - | | - | | - | - |
| 5605 | X | - | | - | | - | - |
| 5624 | X | - | | - | | - | - |
| 5644 | - | - | | - | | X | - |
| 5647 | X | - | | - | | - | - |
| 5816 | X | - | | - | | - | - |
| 5817 | X | - | | - | | - | - |
| 5818 | X | - | | - | | - | X |
| 5819 | X | - | | - | | - | - |
| 5821 | X | X | | - | | X | - |
| 5822 | X | - | | - | | X | - |
| 5823 | X | - | | - | | - | - |
| 5824 | X | - | | - | | - | - |
| 5825 | X | X | | - | | - | - |
| 5826 | X | - | | - | | - | - |
| 5828 | - | X | | - | | - | - |
| 5829 | X | - | | - | | - | - |
| 5830 | X | - | | - | | - | - |
| 5831 | X | - | | - | | - | X |
| 5832 | - | X | | - | | - | - |
| 5833 | - | X | | - | | - | - |
| 5834 | X | - | | - | | - | - |
| 5835 | X | - | | - | | - | - |
| 5836 | X | - | | - | | - | - |
| 5837 | X | - | | - | | - | - |
| 5838 | X | - | | - | | - | - |
| 5839 | X | - | | - | | - | X |
| 5840 | X | - | | - | | X | - |
| 5841 | X | X | | - | | - | - |
| 5842 | X | - | | - | | X | - |
| 5843 | X | - | | - | | - | - |
| 5844 | X | X | | - | | - | - |
| 5845 | X | X | | - | | - | - |
| 5846 | X | X | | - | | - | - |
| 5847 | X | - | | - | | - | - |
| 5848 | - | X | | - | | - | - |
| 5849 | X | - | | - | | - | - |
| 5850 | X | - | | - | | - | - |
| 5852 | X | - | | - | | - | - |
| 5854 | X | - | | - | | - | - |
| 5855 | X | - | | - | | - | - |
| 5856 | X | - | | - | | - | - |
| 5857 | X | X | | - | | - | - |
| 5858 | - | X | | - | | - | - |
| 5860 | X | - | | - | | - | - |
| 5861 | X | - | | - | | X | - |
| 5862 | X | - | | - | | - | X |
| 5863 | - | X | | - | | - | - |
| 5865 | - | X | | - | | - | - |
| 5866 | X | - | | X | | - | - |
| 5867 | X | - | | - | | - | - |
| 5868 | X | - | | - | | X | - |
| 5870 | - | X | | - | | - | - |
| 5871 | - | X | | - | | - | - |
| 5872 | X | - | | - | | - | - |
| 5875 | X | - | | - | | - | - |
| 5876 | X | - | | - | | - | - |
| 5877 | - | X | | - | | - | - |
| 5878 | X | - | | - | | - | - |
| 5879 | X | - | | - | | - | - |
| 5880 | X | - | | - | | - | - |
| 5881 | X | - | | - | | - | - |
| 5882 | X | - | | - | | - | - |
| 5884 | X | - | | - | | - | - |
| 5885 | X | - | | - | | - | - |
| 5886 | X | - | | - | | - | - |
| 5887 | X | - | | - | | - | - |
| 5889 | X | - | | - | | - | - |
| 5890 | X | - | | - | | - | - |
| 5891 | X | - | | - | | - | X |
| 5892 | X | - | | - | | - | - |
| 5893 | X | - | | - | | - | - |
| 5894 | X | - | | - | | - | - |
| 5895 | X | - | | - | | - | - |
| 5897 | X | - | | - | | - | - |
| 5899 | X | - | | - | | - | - |
| 5902 | X | - | | - | | - | - |
| 5903 | X | - | | - | | - | - |
| 5904 | X | - | | - | - | | - |
| 5920 | X | - | | - | - | | - |
| 5921 | X | X | | - | - | | - |
| 5923 | X | - | | - | - | | - |
| 5924 | X | - | | - | - | | - |
| 5925 | X | - | | - | X | | - |
| 5928 | X | - | | - | - | | - |
| 5930 | X | - | | - | - | | - |
| 5931 | X | X | | - | - | | - |
| 5932 | X | X | | - | - | | - |
| 5933 | X | - | | - | - | | - |
| 5934 | X | - | | - | - | | - |
| 5935 | X | - | | - | - | | - |
| 5936 | X | X | | - | - | | - |
| 5937 | - | X | | - | - | | - |
| 5938 | X | - | | - | - | | - |
| 5939 | X | - | | - | - | | - |
| 5940 | X | X | | - | - | | - |
| 5941 | X | - | | - | - | | - |
| 5942 | X | - | | - | - | | - |
| 5943 | X | - | | - | - | | - |
| 5944 | X | - | | - | X | | - |
| 5945 | X | - | | - | - | | - |
| 5946 | X | - | | - | - | | - |
| 5947 | X | - | | - | - | | - |
| 5948 | X | - | | - | - | | - |
| 5950 | X | - | | - | - | | - |
| 5951 | X | - | | - | - | | - |
| 5952 | X | - | | - | - | | - |
| 5953 | X | - | | - | - | | - |
| 5954 | X | - | | - | - | | - |
| 5955 | X | - | | - | - | | - |
| 5956 | X | - | | - | - | | - |
| 5957 | X | - | | - | - | | - |
| 5958 | - | X | | - | - | | - |
| 5959 | X | X | | - | X | | - |
| 5960 | X | X | | - | - | | - |
| 5961 | - | X | | - | - | | - |
| 5962 | X | - | | - | - | | - |
| 5964 | X | X | | - | - | | - |
| 5965 | X | - | | - | - | | - |
| 5966 | X | - | | - | - | | - |
| 5967 | X | - | | - | - | | - |
| 5968 | X | - | | - | - | | - |
| 5969 | X | - | | - | X | | - |
| 5970 | X | - | | - | - | | - |
| 5971 | X | - | | - | - | | - |
| 5972 | X | - | | - | - | | - |
| 5973 | X | - | | - | - | | - |
| 5974 | X | - | | - | - | | - |
| 5975 | X | - | | - | - | | - |
| 5978 | X | - | | - | - | | - |
| 5979 | X | - | | - | - | | - |
| 5980 | X | X | | - | - | | - |
| 5981 | X | X | | - | X | | - |
| 5982 | X | - | | - | - | | - |
| 5984 | X | - | | - | X | | - |
| 5985 | X | - | | - | - | | - |
| 5986 | X | X | | - | X | | - |
| 5987 | X | - | | - | - | | - |
| 5988 | X | - | | - | - | | - |
| 5989 | X | - | | - | - | | - |
| 5990 | X | - | | - | - | | - |
| 5991 | X | - | | - | - | | - |
| 5992 | X | - | | - | - | | - |
| 5993 | - | - | | X | X | | - |
| 5994 | - | X | | - | - | | - |
| 5995 | X | - | | - | X | | - |
| 5996 | - | X | | - | - | | - |
| 5997 | X | - | | - | - | | - |
| 5998 | X | - | | - | - | | - |
| 6000 | X | - | | - | - | | - |
| 6001 | X | - | | - | - | | - |
| 6003 | X | - | | - | - | | - |
| 6004 | X | - | | - | X | | - |
| 6005 | X | - | | - | - | | - |
| 6006 | X | - | | - | - | | - |
| 6023 | X | - | | X | - | | - |
| 6024 | X | X | | - | - | | - |
| 6026 | X | - | | - | X | | - |
| 6027 | X | - | | - | - | | - |
| 6028 | X | - | | - | - | | - |
| 6029 | X | - | | - | X | | - |
| 6030 | X | - | | - | - | | - |
| 6031 | X | - | | - | - | | - |
| 6032 | X | - | | - | - | | - |
| 6033 | X | - | | - | - | | - |
| 6034 | X | - | | - | X | | - |
| 6035 | X | - | | - | - | | - |
| 6036 | X | - | | - | - | | - |
| 6037 | X | - | | - | - | | - |
| 6038 | X | - | | - | - | | - |
| 6039 | X | - | | - | - | | - |
| 6040 | X | - | | - | - | | - |
| 6041 | X | - | | - | - | | - |
| 6042 | X | - | | - | - | | X |
| 6044 | X | - | | - | - | | - |
| 6046 | X | X | | - | X | | - |
| 6047 | X | - | | - | - | | - |
| 6048 | X | - | | - | - | | - |
| 6049 | X | - | | - | - | | - |
| 6050 | X | - | | - | - | | - |
| 6051 | X | - | | - | - | | - |
| 6052 | X | X | | - | - | | - |
| 6053 | X | - | | - | - | | - |
| 6055 | X | - | | - | - | | - |
| 6056 | X | - | | - | - | | - |
| 6057 | - | X | | - | - | | - |
| 6058 | X | X | | X | X | | - |
| 6059 | X | - | | - | - | | - |
| 6060 | - | X | | - | - | | - |
| 6061 | X | - | | - | - | | - |
| 6062 | X | - | | - | - | | - |
| 6066 | X | - | | - | - | | - |
| 6067 | X | - | | - | - | | - |
| 6068 | X | - | | - | - | | - |
| 6069 | X | - | | - | X | | - |
| 6071 | X | - | | - | - | | - |
| 6073 | X | - | | - | X | | - |
| 6074 | - | X | | - | X | | - |
| 6075 | X | - | | - | - | | - |
| 6078 | X | - | | - | - | | - |
| 6081 | X | - | | - | - | | - |
| 6111 | X | - | | - | X | | - |
| 6378 | - | X | | - | - | | - |
| 6379 | X | X | | - | - | | - |
| 6380 | X | - | | - | - | | - |
| 6382 | - | X | | - | - | | - |
| 6383 | X | - | | - | - | | - |
| 6384 | X | - | | - | X | | - |
| 6385 | - | X | | - | - | | - |
| 6386 | X | - | | - | - | | - |
| 6387 | X | - | | - | X | | - |
| 6388 | X | - | | - | - | | - |
| 6389 | X | - | | - | - | | - |
| 6390 | X | X | | - | - | | - |
| 6391 | - | - | | - | X | | - |
| 6392 | X | - | | - | - | | - |
| 6393 | X | - | | - | - | | - |
| 6394 | - | - | | - | X | | - |
| 6395 | X | - | | - | - | | - |
| 6396 | X | - | | - | - | | - |
| 6397 | X | - | | - | - | | - |
| 6398 | X | - | | - | - | | - |
| 6399 | X | - | | - | - | | - |
| 6400 | X | - | | - | - | | X |

Key: * = Basket Number; X = Present; - = Absent.
