# Supplementary material for: Skeletal Remains from Punic Carthage Do Not Support Systematic Sacrifice of Infants
Source: PLoS One. 2010 Feb 17;5(2):e9177. doi: 10.1371/journal.pone.0009177 (PMC2822869; doi:10.1371/journal.pone.0009177)
Supplement: Table S2 — Demographic Profile of Human Remains, Carthaginian Tophet. (0.93 MB DOC) [file pone.0009177.s002.doc]

| **Urn** | **Phase** | **Tanit** | **Area** | **Individual** | **Sex** | **Suggested Dental Age** | | **Neonatal Line** | |
| --- | --- | --- | --- | --- | --- | --- | --- | --- | --- |
| *2 |  |  |  | 1 |  | Birth | |  | |
| *2 |  |  |  | 2 |  | 3 Months | |  | |
| *3 |  |  |  | 1 |  | 2 to 3 Months | |  | |
| *4 |  |  |  | 1 |  | 5 Months | |  | |
| *6 |  |  |  | 1 |  | Fetal | |  | |
| *6 |  |  |  | 2 |  | 4 to 5 Years | |  | |
| *7 |  |  |  | 1 |  |  | |  | |
| *9 |  |  |  | 1 |  | Fetal | |  | |
| *9 |  |  |  | 2 |  | 5 Months | |  | |
| *10 |  |  |  | 1 |  | ~Birth | |  | |
| *10 |  |  |  | 2 |  | 1 to 2 Months | |  | |
| *10 |  |  |  | 3 |  | 10 Months | |  | |
| *20 |  |  |  | 1 | Male | 3 Months | |  | |
| *20 |  |  |  | 2 |  | <5 Months | |  | |
| *21 |  |  |  | 1 |  | 1 to 2 Months | |  | |
| *21 |  |  |  | 2 |  | 6 Months | |  | |
| *27 |  |  |  | 1 |  | 5 Months | |  | |
| *27 |  |  |  | 2 |  | <5 Months | |  | |
| *33 |  |  |  | 1 |  | 1 to 2 Months | |  | |
| *36 |  |  |  | 1 | Female | ~Birth | |  | |
| *36 |  |  |  | 1 |  |  | |  | |
| *40 |  |  |  | 1 |  | 2 Months | |  | |
| *40 |  |  |  | 2 |  | 2 to 3 Months | |  | |
| *46 |  |  |  | 1 |  |  | |  | |
| *108 |  |  |  | 1 |  | ≤Birth | |  | |
| *131 |  |  |  | 1 |  |  | |  | |
| *156 |  |  |  | 1 |  | Late Third Trimester | |  | |
| *168 |  |  |  | 1 |  |  | |  | |
| 1506 | 6+ | III | 2 | 1 |  | Late Third Trimester | |  | |
| 2757 |  |  |  | 1 |  | ~Birth | |  | |
| 2844 | 6+ | III | 1 | 1 |  | ≤Birth | |  | |
| 2844 | 6+ | III | 1 | 2 |  | 3 Months | |  | |
| 2845 | 5 | II | 1 | 1 |  | 1 to 2 Months | |  | |
| 2845 | 5 | II | 1 | 2 |  | 7 to 8 Months | |  | |
| 2846 | 6 | II | 1 | 1 | Female | ~Birth | |  | |
| 2848 | 5 | II | 1 | 1 | Female | 5 Months | |  | |
| 2848 | 5 | II | 1 | 2 |  | 2.5 to 3 Years | |  | |
| 3091 | 7 | III | 1 | 1 |  | Late Third Trimester | |  | |
| 3091 | 7 | III | 1 | 2 |  | 4 Months | |  | |
| 3092 | 7 | III | 1 | 1 |  | Late Third Trimester | |  | |
| 3093 | 7 | III | 1 | 1 |  | 1 to 2 Months | |  | |
| 3093 | 7 | III | 1 | 2 |  | 1 to 2 Months | |  | |
| 3156 | 7 | III | 1 | 1 |  | 11 Months | |  | |
| 3158 | 6 | II | 1 | 1 |  | 3 Months | |  | |
| 3158 | 6 | II | 1 | 2 |  | <3 Years | |  | |
| 3159 | 7 | III | 1 | 1 |  | 1 to 2 Months | | Present | |
| 3159 | 7 | III | 1 | 2 |  | | 1 to 2 Months | |  |
| 3160 | 7 | III | 1 | 1 |  | | <Birth | |  |
| 3163 | 6 | II | 1 | 1 |  | | Late Third Trimester | | Present |
| 3164 | 6 | II | 1 | 1 |  | | Late Third Trimester | |  |
| 3164 | 6 | II | 1 | 2 |  | | ≤Birth | |  |
| 3164 | 6 | II | 1 | 3 |  | | ≤Birth | |  |
| 3165 | 6 | II | 1 | 1 | Indeterminate | | 2 Months | |  |
| 3167 | 6 | II | 1 | 1 |  | | ~Birth | | Present |
| 3168 | 7 | III | 1 | 1 |  | | ≤Birth | |  |
| 3169 | 7 | III | 1 | 1 |  | | 1 to 2 Months | |  |
| 3171 | 7 | III | 1 | 1 |  | | Late Third Trimester | |  |
| 3171 | 7 | III | 1 | 2 |  | | <Birth | |  |
| 3171 | 7 | III | 1 | 3 |  | | 2 Years | |  |
| 3176 | 7 | III | 1 | 1 |  | | <Birth | |  |
| 3176 | 7 | III | 1 | 2 |  | | 3 Months | |  |
| 3176 | 7 | III | 1 | 3 |  | | 4 Months | |  |
| 3177 | 6+ | III | 3 | 1 |  | | 3 Months | |  |
| 3177 | 6+ | III | 3 | 1 |  | |  | |  |
| 3178 | 6 | II | 1 | 1 |  | | ≤Birth | | Absent |
| 3179 | 6 | II | 1 | 1 | Male | | ~Birth | |  |
| 3179 | 6 | II | 1 | 2 | Male | | 9 Months | |  |
| 3180 | 7 | III | 1 | 1 |  | | ~Birth | |  |
| 3180 | 7 | III | 1 | 1 |  | |  | |  |
| 3185 | 6 | II | 1 | 1 |  | | 8 to 9 Months | |  |
| 3186 | 7 | III | 1 | 1 |  | | 6 Months | |  |
| 3186 | 7 | III | 1 | 2 |  | | 6 Months | |  |
| 3186 | 7 | III | 1 | 3 |  | | 8 Months | |  |
| 3187 | 6 | II | 1 | 1 |  | | 2 to 3 Months | |  |
| 3187 | 6 | II | 1 | 2 |  | | 2 to 3 Months | |  |
| 3187 | 6 | II | 1 | 3 |  | | <1 year | |  |
| 3190 | 6 | II | 1 | 1 |  | | Birth | |  |
| 3191 | 6 | II | 1 | 1 |  | | Late Third Trimester | |  |
| 3191 | 6 | II | 1 | 2 |  | | <11 Months | |  |
| 3193 |  |  |  | 1 |  | | 1 to 2 Months | |  |
| 4436 | 6+ | III | 1 | 1 |  | | Fetal | |  |
| 4438 | 5+ | II | 2 | 1 |  | | Fetal | |  |
| 4612 | 7 | III | 1 | 1 | Female | | 2 to 3 Months | |  |
| 4612 | 7 | III | 1 | 1 |  | |  | |  |
| 4613 | 7 | III | 1 | 1 |  | | 5 to 6 Years | |  |
| 4614 | 7 | III | 1 | 1 |  | | Fetal | |  |
| 4614 | 7 | III | 1 | 2 |  | | Fetal | |  |
| 4939 | 6+ | III | 1 | 1 | Female | |  | |  |
| 4941 | 6 | II | 1 | 1 |  | |  | |  |
| 4942 | 6 | II | 1 | 1 |  | | Late Third Trimester | |  |
| 4942 | 6 | II | 1 | 2 |  | | 10 to 11 Months | |  |
| 4957 | 6+ | III | 4 | 1 |  | |  | |  |
| 4957 | 6+ | III | 4 | 2 |  |  | |  | |
| 4957 | 6+ | III | 4 | 3 |  |  | |  | |
| 4957 | 6+ | III | 4 | 4 |  |  | |  | |
| 5007 |  |  |  | 1 |  | Fetal | |  | |
| 5090 |  |  |  | 1 |  |  | |  | |
| 5138 | 5+ | II | 2 | 1 |  | ~Birth | |  | |
| 5138 | 5+ | II | 2 | 2 |  | ~Birth | |  | |
| 5138 | 5+ | II | 2 | 3 |  | 1 to 2 Months | |  | |
| 5163 |  |  |  | 1 |  | ≤Birth | | Absent | |
| 5170 |  |  |  | 4 |  | ≤Birth | |  | |
| 5171 | 5+ | II | 2 | 1 |  | Fetal | |  | |
| 5172 | 5 | II | 2 | 1 |  | ~Birth | |  | |
| 5173 | 6 | II | 1 | 1 |  | Fetal | |  | |
| 5173 | 6 | II | 1 | 2 |  | Fetal | |  | |
| 5173 | 6 | II | 1 | 3 |  | ≤Birth | |  | |
| 5173 | 6 | II | 1 | 4 |  | ≤Birth | |  | |
| 5173 | 6 | II | 1 | 5 |  | 2 to 3 Months | |  | |
| 5189 | 5 | II | 3 | 1 |  | ≤Birth | |  | |
| 5190 | 5 | II | 1 | 1 |  | 3 to 4 Years | |  | |
| 5191 | 7 | III | 1 | 1 |  | 6 Months | |  | |
| 5407 | 6 | II | 1 | 1 |  | 3 Months | |  | |
| 5409 | 4 | II | 1 | 1 |  | Late Third Trimester | |  | |
| 5410 | 4 | II | 2 | 1 | Female | ~Birth | | Absent | |
| 5412 | 7 | III | 1 | 1 |  |  | |  | |
| 5414 | 5 | II | 2 | 1 | Female | Fetal | |  | |
| 5414 | 5 | II | 2 | 2 | Female |  | |  | |
| 5415 | 4 | II | 2 | 1 |  |  | |  | |
| 5415 | 4 | II | 2 | 1 |  | <Birth | |  | |
| 5416 | 5 | II | 2 | 1 |  | ~Birth | |  | |
| 5416 | 5 | II | 2 | 1 |  |  | |  | |
| 5417 | 7 | II | 2 | 1 |  |  | |  | |
| 5417 | 7 | II | 2 | 1 |  | Late Third Trimester | |  | |
| 5498 |  |  |  | 1 |  | Late Third Trimester | |  | |
| 5510 |  |  |  | 2 |  | 2 Months | |  | |
| 5516 | 7 | III | 4 | 1 |  | Fetal | |  | |
| 5516 | 7 | III | 4 | 2 |  | Fetal | |  | |
| 5517 | 7 | III | 4 | 1 |  | <Birth | |  | |
| 5517 | 7 | III | 4 | 2 |  | 1 to 2 Months | |  | |
| 5519 | 5+ | II | 2 | 1 |  | Fetal | |  | |
| 5525 | 6 | II | 1 | 1 |  | 3 to 4 Years | |  | |
| 5528 | 6 | II | 1 | 1 |  |  | |  | |
| 5529 | 7 | III | 1 | 1 | Male | Fetal | |  | |
| 5529 | 7 | III | 1 | 2 |  | Fetal | |  | |
| 5531 | 5 | II | 2 | 1 |  | ~Birth | | Present | |
| 5533 | 8 | III | 4 | 1 |  | Fetal | |  | |
| 5533 | 8 | III | 4 | 2 |  | <Birth | |  | |
| 5533 | 8 | III | 4 | 3 |  | 3 to 5 Months | |  | |
| 5537 | 6 | II | 3 | 1 |  | ~Birth | |  | |
| 5537 | 6 | II | 3 | 2 |  | 3 to 4 Months | |  | |
| 5537 | 6 | II | 3 | 3 |  | 9 Months | |  | |
| 5538 | 6 | II | 3 | 1 | Male | Fetal | |  | |
| 5541 | 5 | II | 1 | 1 |  | Birth | |  | |
| 5541 | 5 | II | 1 | 2 |  | 1 to 2 Months | |  | |
| 5542 | 5 | II | 1 | 1 |  | Fetal to Term | |  | |
| 5542 | 5 | II | 1 | 2 |  | 1 to 2 Months | |  | |
| 5542 | 5 | II | 1 | 3 |  | 3 Months | |  | |
| 5543 | 4 | II | 2 | 1 |  | Late Third Trimester | |  | |
| 5543 | 4 | II | 2 | 2 |  | 6 to 7 Months | |  | |
| 5543 | 4 | II | 2 | 3 |  | 2.5 to 3 Years | |  | |
| 5544 | 5 | II | 2 | 1 |  |  | |  | |
| 5545 | 5 | II | 2 | 1 | Female |  | |  | |
| 5546 | 6 | II | 2 | 1 |  |  | |  | |
| 5547 | 5 | II | 2 | 1 |  | Fetal | |  | |
| 5550 | 4 | II | 3 | 1 |  | Fetal to Term | |  | |
| 5550 | 4 | II | 3 | 2 |  | <Birth | |  | |
| 5550 | 4 | II | 3 | 3 |  | 5 to 6 Months | |  | |
| 5551 | 7 | III | 4 | 1 |  | Fetal to Term | |  | |
| 5551 | 7 | III | 4 | 2 |  | 5 Months | |  | |
| 5552 | 7 | III | 4 | 1 |  | Fetal | |  | |
| 5552 | 7 | III | 4 | 2 |  | 9 Months | |  | |
| 5553 | 7 | III | 4 | 1 |  | ≤Birth | |  | |
| 5555 | 5 | II | 1 | 1 |  | Birth | |  | |
| 5558 | 6 | II | 1 | 1 |  | ~Birth | |  | |
| 5559 | 6 | II | 1 | 1 |  | Third Trimester | |  | |
| 5559 | 6 | II | 1 | 2 |  | Birth to 1 month | |  | |
| 5559 | 6 | II | 1 | 3 |  | 6 Months (intrusive) | |  | |
| 5561 | 4 | II | 2 | 1 |  | ≤Birth | |  | |
| 5561 | 4 | II | 2 | 2 |  | <Birth | |  | |
| 5561 | 4 | II | 2 | 3 |  | <5 Months | |  | |
| 5563 | 6 | II | 1 | 1 |  |  | |  | |
| 5565 | 6 | II | 1 | 1 |  | Fetal | |  | |
| 5565 | 6 | II | 1 | 2 |  | ≤Birth | |  | |
| 5565 | 6 | II | 1 | 3 |  | 2 Months | |  | |
| 5567 | 6 | II | 1 | 1 |  | ≤Birth | |  | |
| 5567 | 6 | II | 1 | 2 |  | ≤Birth | |  | |
| 5567 | 6 | II | 1 | 3 |  | 1 to 2 Months | |  | |
| 5568 | 5 | II | 3 | 1 |  | ~ Birth | |  | |
| 5569 | 6 | II | 1 | 1 |  | ≤Birth | |  | |
| 5570 | 5 | II | 3 | 1 |  | ~Birth | | Present | |
| 5571 | 4 | II | 1 | 1 |  | Late Third Trimester | |  | |
| 5571 | 4 | II | 1 | 2 |  | 8 Months | |  | |
| 5573 | 5 | II | 1 | 1 |  | Birth | |  | |
| 5573 | 5 | II | 1 | 2 |  | | 4 Months | |  |
| 5574 | 6 | II | 2 | 1 |  | |  | |  |
| 5575 | 5 | II | 2 | 1 |  | | Fetal | |  |
| 5575 | 5 | II | 2 | 2 |  | | 8 Months | |  |
| 5576 | 5 | II | 1 | 1 |  | | Late Third Trimester | | Absent |
| 5577 | 4 | II | 2 | 1 | Female | | Fetal to Term | |  |
| 5578 | 5 | II | 2 | 1 |  | | ~Birth | |  |
| 5578 | 5 | II | 2 | 2 |  | | <6 Months | |  |
| 5579 | 5 | II | 2 | 1 |  | |  | |  |
| 5579 | 5 | II | 2 | 1 | Indeterminate | |  | |  |
| 5580 | 5 | II | 2 | 1 |  | | Fetal | |  |
| 5581 | 5 | II | 3 | 1 |  | | 3 Months | |  |
| 5582 | 5 | II | 1 | 1 |  | | ≤Birth | | Absent |
| 5586 | 6 | II | 1 | 1 |  | | ~Birth | |  |
| 5586 | 6 | II | 1 | 2 |  | | 2 Months | |  |
| 5586 | 6 | II | 1 | 3 |  | | 9 to 10 Months | |  |
| 5586 | 6 | II | 1 | 4 |  | | 6 Years | |  |
| 5587 | 4 | II | 2 | 1 |  | | Late Third Trimester | | Present |
| 5588 | 5 | II | 3 | 1 |  | | Birth | |  |
| 5588 | 5 | II | 3 | 2 |  | | Birth | |  |
| 5588 | 5 | II | 3 | 3 |  | | 5 to 6 Months | |  |
| 5589 | 5 | II | 3 | 1 |  | | 3 to 4 Months | |  |
| 5589 | 5 | II | 3 | 2 |  | | 3 Months | |  |
| 5590 | 5 | II | 3 | 1 |  | | Late Third Trimester | |  |
| 5590 | 5 | II | 3 | 2 |  | | Late Third Trimester | |  |
| 5590 | 5 | II | 3 | 3 |  | | 6 to 7 Months | |  |
| 5591 | 8 | III | 4 | 1 |  | |  | |  |
| 5592 |  |  |  | 1 |  | |  | |  |
| 5592 |  |  |  | 2 |  | |  | |  |
| 5593 | 4 | II | 1 | 1 |  | | <2 Years | |  |
| 5594 | 6 | II | 1 | 1 |  | | <Birth | |  |
| 5594 | 6 | II | 1 | 2 |  | | 6 Months | |  |
| 5595 | 7 | III | 1a | 1 |  | |  | |  |
| 5596 | 5 | II | 2 | 1 |  | | 3 Years | |  |
| 5597 | 5 | II | 3 | 1 |  | |  | |  |
| 5598 | 6 | II | 3 | 1 |  | | Fetal | |  |
| 5598 | 6 | II | 3 | 2 |  | | ~Birth | |  |
| 5598 | 6 | II | 3 | 3 |  | | 2 to 3 Months | |  |
| 5598 | 6 | II | 3 | 4 |  | | 6 Months | |  |
| 5598 | 6 | II | 3 | 5 |  | | ≤1 Year | |  |
| 5599 | 5 | II | 3 | 1 |  | | Birth | | Present |
| 5599 | 5 | II | 3 | 2 |  | | Birth | |  |
| 5602 | 4 | II | 1 | 1 |  | | Late Third Trimester | |  |
| 5602 | 4 | II | 1 | 2 |  | | 5 Months | |  |
| 5603 | 4 | II | 1 | 1 |  | | Late Third Trimester | |  |
| 5603 | 4 | II | 1 | 2 |  | | 4 to 5 Months | |  |
| 5604 | 4 | II | 1 | 1 |  | | ~Birth | |  |
| 5604 | 4 | II | 1 | 2 |  | | 8 Months | |  |
| 5623 | 5 | II | 3 | 1 | Male | | Late Third Trimester | |  |
| 5623 | 5 | II | 3 | 2 | Female | |  | |  |
| 5625 | 6 | II | 1 | 1 |  | | 2 Months | | Present |
| 5647 | 5 | II | 1 | 1 |  | |  | |  |
| 5788 |  |  |  | 1 |  | | Third Trimester | |  |
| 5815 | 7 | III | 1 | 1 |  | | Birth to 3 Months | |  |
| 5816 | 7 | III | 1 | 1 |  | | 9 to 10 Months | |  |
| 5817 | 6 | II | 1 | 1 | Indeterminate | | Late Third Trimester | | Present |
| 5817 | 6 | II | 1 | 2 |  | | ~Birth | |  |
| 5817 | 6 | II | 1 | 3 |  | | 2 Months | |  |
| 5818 | 5 | II | 1 | 1 | Female | | Late Third Trimester | | Absent |
| 5821 | 6 | II | 1 | 1 |  | | 2 Months | |  |
| 5822 | 5 | II | 1 | 1 |  | | ~Birth | |  |
| 5822 | 5 | II | 1 | 2 |  | | 4 Years | |  |
| 5823 | 4 | II | 1 | 1 |  | | <Birth | |  |
| 5824 | 5 | II | 1 | 1 | Female | | ~Birth | |  |
| 5824 | 5 | II | 1 | 2 |  | | 1 to 2 Months | |  |
| 5826 | 6 | II | 1 | 1 |  | | 8 Months | |  |
| 5827 | 6 | II | 1 | 1 | Female | | Late Third Trimester | |  |
| 5827 | 6 | II | 1 | 2 |  | | <Birth | |  |
| 5827 | 6 | II | 1 | 3 |  | | 3 Months | |  |
| 5829 | 6 | II | 1 | 1 |  | | <Birth | | Present |
| 5830 | 6 | II | 1 | 1 | Female | | Late Third Trimester | |  |
| 5830 | 6 | II | 1 | 2 |  | | 10 to 11 Months | |  |
| 5831 | 5 | II | 1 | 1 |  | | Late Third Trimester | | Absent |
| 5831 | 5 | II | 1 | 2 |  | | 10 Months | |  |
| 5834 | 4 | II | 1 | 1 |  | | ≤Birth | | Absent |
| 5835 | 4 | II | 1 | 1 | Female | | ≤Birth | |  |
| 5835 | 4 | II | 1 | 2 |  | | 5 Months | |  |
| 5835 | 4 | II | 1 | 3 |  | | 1.5 Years | |  |
| 5836 | 3 | I | 1 | 1 |  | | 2 to 3 Months | |  |
| 5836 | 3 | I | 1 | 2 |  | | 5 to 6 Months | |  |
| 5837 | 3 | I | 1 | 1 |  | | 1 to 2 Months | |  |
| 5838 | 3 | I | 1 | 1 |  | | ≤Birth | |  |
| 5839 | 3 | I | 1 | 1 |  | | Late Third Trimester | |  |
| 5839 | 3 | I | 1 | 2 |  | | ~Birth | |  |
| 5840 | 3 | I | 1 | 1 |  | | Late Third Trimester | | Absent |
| 5841 | 4 | II | 1 | 1 |  | |  | |  |
| 5841 | 4 | II | 1 | 1 | Female | |  | |  |
| 5842 | 3 | I | 1 | 1 |  | | ≤Birth | |  |
| 5842 | 3 | I | 1 | 2 |  | | 2 Months | |  |
| 5843 | 5 | II | 1 | 1 | Indeterminate | | 2 Months | |  |
| 5844 | 3 | I | 1 | 1 |  | | ~Birth | |  |
| 5845 | 3 | I | 1 | 1 |  | | ~Birth | |  |
| 5847 | 1 | I | 1 | 1 |  | | 5 to 6 Months | |  |
| 5847 | 1 | I | 1 | 2 |  | | 9 Months | |  |
| 5849 | 4 | II | 1 | 1 | Female | | 2 to 3 Months | |  |
| 5850 | 5 | II | 1 | 1 | Female | | 1 Month | |  |
| 5850 | 5 | II | 1 | 2 | Male | | 1 Month | |  |
| 5850 | 5 | II | 1 | 3 |  | | 1 Month | |  |
| 5852 | 6 | II | 1 | 1 |  | | Birth | | Present |
| 5853 | 6 | II | 1 | 1 |  | | Fetal to Term | |  |
| 5853 | 6 | II | 1 | 2 |  | | ≤Birth | |  |
| 5853 | 6 | II | 1 | 3 |  | | 2 to 3 Months | |  |
| 5854 | 6 | II | 1 | 1 |  | | 1 to 2 Months | |  |
| 5855 | 5 | II | 1 | 1 |  | | Late Third Trimester | | Absent |
| 5856 | 3 | I | 1 | 1 |  | | Late Third Trimester | |  |
| 5857 | 3 | I | 1 | 1 |  | | 1 to 2 Months | |  |
| 5857 | 3 | I | 1 | 2 |  | | 5 to 6 Years | |  |
| 5861 | 3 | I | 1 | 1 |  | |  | |  |
| 5862 | 3 | I | 1 | 1 |  | | ≤Birth | | Present |
| 5862 | 3 | I | 1 | 2 |  | | 5 Months | |  |
| 5866 | 6 | II | 1 | 1 |  | | Late Third Trimester | |  |
| 5867 | 6 | II | 1 | 1 |  | | ≤Birth | |  |
| 5868 | 3 | I | 1 | 1 |  | | ≤Birth | | Present |
| 5869 | 6 | II | 1 | 1 |  | | Fetal | |  |
| 5869 | 6 | II | 1 | 2 |  | | Fetal to Term | |  |
| 5872 | 4 | II | 1 | 1 |  | | 7 to 8 Months | |  |
| 5872 | 4 | II | 1 | 2 |  | | 7 to 8 Months | |  |
| 5874 | 4 | II | 1 | 1 |  | |  | |  |
| 5875 | 3 | I | 1 | 1 |  | |  | |  |
| 5876 | 3 | I | 1 | 1 |  | | 4 Months | |  |
| 5880 | 1 | I | 1 | 1 |  | | Late Third Trimester | | Absent |
| 5881 | 6 | II | 1 | 1 | Male | | 1 to 2 Months | |  |
| 5882 | 1 | I | 1 | 1 |  | |  | |  |
| 5883 | 6 | II | 1 | 1 | Female | | Late Third Trimester | | Present |
| 5884 | 6 | II | 1 | 1 |  | |  | |  |
| 5885 | 7 | III | 1 | 1 |  | | ≤Birth | |  |
| 5885 | 7 | III | 1 | 2 |  | | 2 Months | |  |
| 5886 | 7 | III | 1 | 1 |  | | 8 Months | |  |
| 5887 | 6 | II | 1 | 1 | Female | | ≤Birth | |  |
| 5887 | 6 | II | 1 | 2 |  | | 2 to 3 Months | |  |
| 5888 | 6 | II | 1 | 1 |  | | Birth | |  |
| 5890 | 5 | II | 1 | 1 | Indeterminate | | ~Birth | |  |
| 5891 | 6 | II | 1 | 1 |  | | ≤Birth | |  |
| 5891 | 6 | II | 1 | 2 |  | | 2 to 3 Months | |  |
| 5892 | 6 | II | 1 | 1 |  | | ≤Birth | |  |
| 5893 | 6 | II | 1 | 1 |  | | Late Third Trimester | |  |
| 5893 | 6 | II | 1 | 2 |  | | <Birth | |  |
| 5894 | 6 | II | 1 | 1 |  | | 2 to 3 Months | |  |
| 5894 | 6 | II | 1 | 2 |  | | 2 to 3 Months | |  |
| 5895 | 6 | II | 1 | 1 | Female | | 3 Months | |  |
| 5895 | 6 | II | 1 | 2 |  | | 3 Months | |  |
| 5895 | 6 | II | 1 | 3 |  | | 10 to 11 Months | |  |
| 5896 | 5 | II | 1 | 1 |  | | ≤Birth | |  |
| 5896 | 5 | II | 1 | 2 |  | | 9 Months | |  |
| 5897 | 3 | I | 1 | 1 |  | | Fetal | |  |
| 5898 | 6 | II | 1 | 1 |  | | Birth | |  |
| 5899 | 6 | II | 1 | 1 |  | | ≤Birth | |  |
| 5899 | 6 | II | 1 | 2 |  | | ≤Birth | |  |
| 5901 | 6 | II | 2 | 1 |  | | 1.5 to 2 Years | |  |
| 5902 | 5 | II | 2 | 1 |  | | ≤Birth | | Absent |
| 5903 | 5 | II | 2 | 1 | Female | | Fetal | |  |
| 5903 | 5 | II | 2 | 2 |  | | Fetal | |  |
| 5903 | 5 | II | 2 | 3 |  | | Fetal to Term | |  |
| 5904 | 5 | II | 2 | 1 |  | | Fetal | |  |
| 5904 | 5 | II | 2 | 2 |  | | 10 to 11 Months | |  |
| 5904 | 5 | II | 2 | 3 |  | | 10 to 11 Months | |  |
| 5904 | 5 | II | 2 | 4 |  | | 2.5 to 3 Years | |  |
| 5919 | 5 | II | 3 | 1 |  | | ≤Birth | |  |
| 5919 | 5 | II | 3 | 2 |  | | ≤1 Year | |  |
| 5920 | 4 | II | 3 | 1 | Female | | Fetal | |  |
| 5920 | 4 | II | 3 | 2 |  | | 4 Months | |  |
| 5922 | 5 | II | 3 | 1 |  | | Late Third Trimester | |  |
| 5923 | 5 | II | 3 | 1 |  | | 4 Months | |  |
| 5923 | 5 | II | 3 | 2 |  | | 7 to 8 Months | |  |
| 5924 | 4 | II | 3 | 1 |  | | Fetal | |  |
| 5924 | 4 | II | 3 | 2 |  | | 3 to 4 Months | |  |
| 5925 | 4 | II | 3 | 1 |  | | Fetal | |  |
| 5925 | 4 | II | 3 | 2 |  | | Fetal to Term | |  |
| 5926 | 4 | II | 3 | 1 |  | | Fetal to Term | |  |
| 5927 | 5 | II | 3 | 1 |  | | Fetal to Term | |  |
| 5927 | 5 | II | 3 | 2 |  | | Fetal to Term | |  |
| 5928 | 5 | II | 3 | 1 |  | | Fetal | |  |
| 5928 | 5 | II | 3 | 2 |  | | 4 to 5 Months | |  |
| 5929 | 5 | II | 3 | 1 |  | | 9 Months | |  |
| 5930 | 5 | II | 3 | 1 |  | | Fetal | |  |
| 5930 | 5 | II | 3 | 2 |  | | Birth | |  |
| 5930 | 5 | II | 3 | 3 |  | | 2 Months | |  |
| 5931 | 5 | II | 3 | 1 |  | | Fetal | |  |
| 5931 | 5 | II | 3 | 2 |  | | Fetal | |  |
| 5932 | 5 | II | 3 | 1 | Female | | Fetal to Term | |  |
| 5932 | 5 | II | 3 | 2 |  | | 1 to 2 Months | |  |
| 5933 | 5 | II | 3 | 1 |  | | 1 to 2 Months | |  |
| 5934 | 6 | II | 3 | 1 |  | | Fetal to Term | |  |
| 5934 | 6 | II | 3 | 2 |  | | 5 to 6 Months | |  |
| 5935 | 6 | II | 3 | 1 |  | | Fetal to Term | |  |
| 5935 | 6 | II | 3 | 2 |  | | Fetal to Term | |  |
| 5935 | 6 | II | 3 | 3 |  | | 4 Months | |  |
| 5938 | 6+ | III | 5 | 1 |  | | 2 to 3 Months | |  |
| 5938 | 6+ | III | 5 | 2 |  | | 4 to 5 Months | |  |
| 5939 | 6+ | III | 5 | 1 |  | | Birth | |  |
| 5939 | 6+ | III | 5 | 2 |  | | 2 to 3 Months | |  |
| 5939 | 6+ | III | 5 | 3 |  | | 9 Months | |  |
| 5940 | 6+ | III | 5 | 1 |  | | 2 to 3 Months | |  |
| 5940 | 6+ | III | 5 | 2 |  | | 6 Months | |  |
| 5940 | 6+ | III | 5 | 3 |  | | 3 to 4 Years | |  |
| 5941 | 6+ | III | 5 | 1 |  | | Fetal to Term | |  |
| 5941 | 6+ | III | 5 | 2 |  | | Fetal to Term | |  |
| 5942 | 6+ | III | 5 | 1 |  | | Fetal | |  |
| 5943 | 6+ | III | 5 | 1 |  | | <6 Months | |  |
| 5943 | 6+ | III | 5 | 2 |  | | ≤1 Year | |  |
| 5944 | 6+ | III | 5 | 1 |  | | Fetal | |  |
| 5944 | 6+ | III | 5 | 2 |  | | 2 to 3 Months | |  |
| 5945 | 5 | II | 5 | 1 | Male | | 2 to 3 Months | |  |
| 5946 | 5 | II | 5 | 1 | Indeterminate | | ≤Birth | |  |
| 5948 | 6+ | III | 5 | 1 | Female | | Birth | | Present |
| 5950 | 6 | II | 5 | 1 |  | | Late Third Trimester | |  |
| 5951 | 3 | I | 5 | 1 |  | | ≤Birth | |  |
| 5951 | 3 | I | 5 | 2 |  | | ≤Birth | |  |
| 5951 | 3 | I | 5 | 3 |  | | 2.5 to 3 Months | |  |
| 5952 | 1 | I | 5 | 1 |  | | Late Third Trimester | | Absent |
| 5953 | 6 | II | 5 | 1 |  | | 4 to 5 Months | |  |
| 5954 | 5 | II | 5 | 1 |  | | Birth | |  |
| 5954 | 5 | II | 5 | 2 |  | | 7 to 8 Months | |  |
| 5955 | 5 | II | 5 | 1 |  | | ≤Birth | |  |
| 5955 | 5 | II | 5 | 2 |  | | Birth to 1 Month | |  |
| 5955 | 5 | II | 5 | 3 |  | | 2 Months | |  |
| 5956 | 6+ | III | 5 | 1 |  | | Birth | |  |
| 5956 | 6+ | III | 5 | 2 |  | | Birth (intrusive?) | |  |
| 5957 | 6+ | III | 5 | 1 |  | | ~1 Month | |  |
| 5959 | 5 | II | 5 | 1 | Female | | Late Third Trimester | |  |
| 5959 | 5 | II | 5 | 2 | Female | | ~Birth | |  |
| 5960 | 5 | II | 5 | 1 |  | | ~Birth | |  |
| 5962 | 5 | II | 5 | 1 | Female | | 5 Months | |  |
| 5963 | 6+ | III | 5 | 1 | Female | |  | |  |
| 5963 | 6+ | III | 5 | 1 |  | |  | |  |
| 5965 | 6+ | III | 5 | 1 |  | | Late Third Trimester | |  |
| 5965 | 6+ | III | 5 | 2 |  | | ≤Birth | |  |
| 5965 | 6+ | III | 5 | 3 |  | | 8 Months | |  |
| 5966 | 6+ | III | 5 | 1 |  | | Birth | | Absent |
| 5967 | 6+ | III | 5 | 1 | Female | | ~Birth | |  |
| 5967 | 6+ | III | 5 | 2 |  | Birth | |  | |
| 5968 | 3 | I | 5 | 1 |  | Fetal to Term | |  | |
| 5968 | 3 | I | 5 | 2 |  | 2 to 3 Months | |  | |
| 5969 | 6+ | III | 5 | 1 |  | 1 Month | |  | |
| 5969 | 6+ | III | 5 | 2 |  | 1 Month | |  | |
| 5970 | 3 | I | 5 | 1 |  | ≤Birth | |  | |
| 5971 | 3 | I | 5 | 1 | Female | Third Trimester | | Absent | |
| 5972 | 5 | II | 5 | 1 |  | 1 Month | |  | |
| 5972 | 5 | II | 5 | 2 |  | 10 Months | |  | |
| 5973 | 2 | I | 5 | 1 |  | 10 to 11 Months | |  | |
| 5974 | 2 | I | 5 | 1 |  | Late Third Trimester | |  | |
| 5974 | 2 | I | 5 | 2 |  | ≤Birth | |  | |
| 5975 | 2 | I | 5 | 1 |  | ≤Birth | |  | |
| 5978 | 6+ | III | 5 | 1 |  | <Birth | |  | |
| 5980 | 1 | I | 5 | 1 |  | 10 to 11 Months | |  | |
| 5981 | 2 | I | 5 | 1 |  | Fetal to Term | |  | |
| 5981 | 2 | I | 5 | 2 |  | 3 to 4 Months | |  | |
| 5982 | 6+ | III | 5 | 1 |  | 2 Months | |  | |
| 5984 | 6 | II | 5 | 2 |  | 9 Months | |  | |
| 5984 | 6 | II | 5 | 3 |  | 2 Years | |  | |
| 5984 | 6 | II | 5 | 4 |  | 5 to 6 Years | |  | |
| 5985 | 3 | I | 5 | 1 |  | Late Third Trimester | |  | |
| 5986 | 5 | II | 5 | 1 |  | Birth | |  | |
| 5986 | 5 | II | 5 | 2 |  | Birth | |  | |
| 5986 | 5 | II | 5 | 3 |  | 3 Months | |  | |
| 5987 | 5 | II | 5 | 1 | Male | ≤Birth | |  | |
| 5987 | 5 | II | 5 | 2 |  | 10 Months | |  | |
| 5988 | 1 | I | 5 | 1 |  | Fetal | |  | |
| 5989 | 1 | I | 5 | 1 |  | ≤Birth | |  | |
| 5989 | 1 | I | 5 | 2 |  | ≤Birth | |  | |
| 5989 | 1 | I | 5 | 3 |  | 3 Months | |  | |
| 5990 | 5 | II | 5 | 1 |  | Late Third Trimester | |  | |
| 5990 | 5 | II | 5 | 2 |  | 9 Months | |  | |
| 5991 | 6+ | III | 5 | 1 | Female | ≤Birth | | Present | |
| 5991 | 6+ | III | 5 | 2 |  | 4 to 5 Months | |  | |
| 5992 | 5 | II | 5 | 1 | Female | 1 to 2 Months | |  | |
| 5995 | 2 | I | 5 | 1 |  | Late Third Trimester | |  | |
| 5995 | 2 | I | 5 | 2 |  | ≤Birth | |  | |
| 5997 | 6+ | III | 5 | 1 |  | 1 to 2 Months | |  | |
| 5997 | 6+ | III | 5 | 2 |  | 3 to 4 Months | |  | |
| 5998 | 5 | II | 5 | 1 |  | ≤Birth | | Absent | |
| 6000 | 6 | II | 5 | 1 | Female | 1 to 2 Months | |  | |
| 6000 | 6 | II | 5 | 2 | Male | <5 Months | |  | |
| 6001 | 6 | II | 5 | 1 |  | 2 to 3 Months | |  | |
| 6001 | 6 | II | 5 | 2 |  | 4 Months | |  | |
| 6003 | 1 | I | 5 | 1 |  | Late Third Trimester | | Absent | |
| 6004 | 1 | I | 5 | 1 |  | <Birth | |  | |
| 6005 | 2 | I | 5 | 1 |  | 8 Months | |  | |
| 6005 | 2 | I | 5 | 2 |  | 9 Months | |  | |
| 6006 | 6 | II | 5 | 1 |  | Late Third Trimester | |  | |
| 6006 | 6 | II | 5 | 2 |  | 5 Months | |  | |
| 6021 |  |  |  | 1 |  | 2 to 3 Months | |  | |
| 6023 | 6+ | III | 6 | 1 |  | ≤Birth | | Present | |
| 6024 | 6+ | III | 6 | 1 |  | Late Third Trimester | |  | |
| 6024 | 6+ | III | 6 | 2 |  | Late Third Trimester | |  | |
| 6024 | 6+ | III | 6 | 3 |  | Late Third Trimester | |  | |
| 6026 | 6+ | III | 6 | 1 |  | Birth | |  | |
| 6027 | 6+ | III | 6 | 1 |  | ~Birth | |  | |
| 6028 | 6+ | III | 6 | 1 |  |  | |  | |
| 6028 | 6+ | III | 6 | 1 | Female |  | |  | |
| 6028 | 6+ | III | 6 | 2 |  |  | |  | |
| 6028 | 6+ | III | 6 | 3 |  |  | |  | |
| 6029 | 6+ | III | 6 | 1 | Male | Late Third Trimester | |  | |
| 6029 | 6+ | III | 6 | 2 |  | Late Third Trimester | |  | |
| 6029 | 6+ | III | 6 | 3 |  | 1 to 2 Months | |  | |
| 6030 | 6+ | III | 6 | 1 |  |  | |  | |
| 6031 | 6+ | III | 6 | 1 |  | ~Birth | |  | |
| 6031 | 6+ | III | 6 | 2 |  | ≤Birth | |  | |
| 6031 | 6+ | III | 6 | 3 |  | 3 Months | |  | |
| 6032 | 6+ | III | 6 | 1 |  | 5 Months | |  | |
| 6033 | 6 | II | 6 | 1 |  | Birth | |  | |
| 6033 | 6 | II | 6 | 2 |  | <Birth | |  | |
| 6034 | 6+ | III | 6 | 1 |  | ≤Birth | |  | |
| 6035 | 6+ | III | 6 | 1 |  | ≤Birth | |  | |
| 6035 | 6+ | III | 6 | 2 |  | 1 to 1.5 Years | |  | |
| 6036 | 6+ | III | 6 | 1 |  | ~Birth | | Absent | |
| 6036 | 6+ | III | 6 | 2 |  | 4 Months | |  | |
| 6037 | 6+ | III | 6 | 1 | Female | Birth to 1 Month | |  | |
| 6038 | 6+ | III | 6 | 1 |  | ~Birth | |  | |
| 6039 | 5? | II | 6 | 1 |  | ≤Birth | |  | |
| 6040 | 5? | II | 6 | 1 |  | Birth | |  | |
| 6040 | 5? | II | 6 | 2 |  | ≤Birth | |  | |
| 6040 | 5? | II | 6 | 3 |  | 2 Months | |  | |
| 6040 | 5? | II | 6 | 4 |  | 4 to 4.5 Years | |  | |
| 6041 | 5? | II | 6 | 1 |  | ≤Birth | |  | |
| 6042 | 3 | I | 6 | 1 |  | ≤Birth | |  | |
| 6042 | 3 | I | 6 | 2 |  | 3 Months | |  | |
| 6043 | 6+ | III | 6 | 1 | Female | Late Third Trimester | |  | |
| 6043 | 6+ | III | 6 | 2 |  | Birth to 1 Month | |  | |
| 6044 | 6+ | III | 6 | 1 |  | 5 to 6 Months | |  | |
| 6045 | 6+ | III | 6 | 1 |  | 9 to 10 Months | |  | |
| 6046 | 6+ | III | 6 | 1 |  | Late Third Trimester | |  | |
| 6046 | 6+ | III | 6 | 2 |  | 5 Months | |  | |
| 6047 | 6+ | III | 6 | 1 |  |  | |  | |
| 6048 | 6+ | III | 6 | 1 |  | 4 Months | |  | |
| 6049 | 6+ | III | 6 | 1 |  | Birth to 1 Month | |  | |
| 6050 | 6+ | III | 6 | 1 |  | ~Birth | |  | |
| 6051 | 6+ | III | 6 | 1 |  | ~Birth | | Absent | |
| 6052 | 6+ | III | 6 | 1 |  | ≤Birth | |  | |
| 6053 | 6+ | III | 6 | 1 |  | 2 Months | |  | |
| 6054 | 6+ | III | 6 | 1 |  | ≤ Birth | | Absent | |
| 6055 | 5? | II | 6 | 1 |  | Late Third Trimester | | Present | |
| 6056 | 6 | II | 6 | 2 |  | ≤Birth | |  | |
| 6056 | 6 | II | 6 | 3 |  | 3 Months | |  | |
| 6056 | 6 | II | 6 | 4 |  | 5 Months | |  | |
| 6058 | 3 | I | 6 | 1 |  | ≤Birth | | Present | |
| 6059 | 1 | I | 6 | 1 |  | Late Third Trimester | |  | |
| 6060 | 2 | I | 6 | 1 |  | 4 Months | |  | |
| 6061 | 6+ | III | 6 | 1 |  | 1 to 1.5 Years | |  | |
| 6062 | 6 | II | 6 | 1 |  | Late Third Trimester | |  | |
| 6062 | 6 | II | 6 | 2 |  | 9 Months | |  | |
| 6063 |  |  |  | 1 |  | Birth to 1 Month | |  | |
| 6064 | 6+ | III | 6 | 1 | Female | 5 Months | |  | |
| 6064 | 6+ | III | 6 | 2 |  | 1.5 Years | |  | |
| 6065 | 3 | I | 6 | 1 |  | Fetal | |  | |
| 6065 | 3 | I | 6 | 2 |  | 2 to 3 Months | |  | |
| 6065 | 3 | I | 6 | 3 |  | 2 to 3 Months | |  | |
| 6065 | 3 | I | 6 | 4 |  | <5 Months | |  | |
| 6065 | 3 | I | 6 | 5 |  | 5 to 6 Months | |  | |
| 6066 | 6+ | III | 6 | 1 |  | 7 to 8 Months | |  | |
| 6067 | 5? | II | 6 | 1 |  | 9 Months | |  | |
| 6068 | 6+ | III | 6 | 1 | Male | ~Birth | | Absent | |
| 6069 | 5? | II | 6 | 1 |  | ~Birth | | Absent | |
| 6069 | 5? | II | 6 | 2 |  | Birth | |  | |
| 6070 |  |  |  | 1 |  | Birth | | Present | |
| 6071 | 6 | II | 6 | 1 |  | Birth to 1 Month | |  | |
| 6073 | 5? | II | 6 | 1 |  | ~Birth | |  | |
| 6073 | 5? | II | 6 | 2 |  | ≤Birth | |  | |
| 6073 | 5? | II | 6 | 3 |  | ≤2 Months | |  | |
| 6075 | 6+ | III | 6 | 1 |  | Late Third Trimester | |  | |
| 6076 | 6+ | III | 6 | 1 |  | 4 Months | |  | |
| 6077 | 5 | II | 6 | 1 |  | Late Third Trimester | |  | |
| 6077 | 5 | II | 6 | 2 |  | 4 Months | |  | |
| 6078 | 6+ | III | 6 | 1 |  | ≤Birth | |  | |
| 6078 | 6+ | III | 6 | 2 |  | 1 to 2 Months | |  | |
| 6079 | 6+ | III | 6 | 1 |  | ~Birth | |  | |
| 6079 | 6+ | III | 6 | 2 |  | Birth | |  | |
| 6079 | 6+ | III | 6 | 3 |  | 2 to 3 Months | |  | |
| 6080 | 6+ | III | 6 | 1 |  | | Late Third Trimester | |  |
| 6080 | 6+ | III | 6 | 2 |  | | 3 to 4 Months | |  |
| 6081 | 5? | II | 6 | 1 |  | | ≤Birth | |  |
| 6081 | 5? | II | 6 | 2 |  | | 1 to 2 Months | |  |
| 6082 | 3 | I | 6 | 1 |  | | Late Third Trimester | |  |
| 6082 | 3 | I | 6 | 2 |  | | 3 Months | |  |
| 6082 | 3 | I | 6 | 3 |  | | 1.5 Years | |  |
| 6083 |  |  |  | 1 |  | | 5 to 6 Months | |  |
| 6111 | 6+ | III | 4 | 1 |  | | <Birth | |  |
| 6379 | 5 | II | 1 | 1 | Female | | ~Birth | |  |
| 6379 | 5 | II | 1 | 2 |  | | 10 to 11 Months | |  |
| 6380 | 3 | I | 1 | 1 |  | | ≤Birth | |  |
| 6383 | 4 | II | 1 | 1 |  | | 9 to 11 Months | |  |
| 6386 | 1 | I | 1 | 1 |  | | ~Birth | |  |
| 6387 | 1 | I | 1 | 1 |  | | ~Birth | |  |
| 6388 | 2 | I | 1 | 1 |  | | Late Third Trimester | |  |
| 6389 | 6 | II | 1 | 1 |  | | Late Third Trimester | |  |
| 6389 | 6 | II | 1 | 2 |  | | ~Birth | |  |
| 6389 | 6 | II | 1 | 3 |  | | >6 Months | |  |
| 6392 | 3 | I | 1 | 1 | Male | | ~Birth | |  |
| 6392 | 3 | I | 1 | 2 |  | | ≤Birth | |  |
| 6392 | 3 | I | 1 | 3 |  | | ≤Birth | |  |
| 6393 | 5 | II | 1 | 1 |  | | Birth | | Absent |
| 6393 | 5 | II | 1 | 2 |  | | Birth | |  |
| 6393 | 5 | II | 1 | 3 |  | | ≤Birth | |  |
| 6393 | 5 | II | 1 | 4 |  | | 5 to 6 Months | |  |
| 6394 | 4 | II | 1 | 1 |  | | 2 to 3 Months | |  |
| 6394 | 4 | II | 1 | 2 |  | | 5 Years | |  |
| 6395 | 3 | I | 1 | 1 |  | | Fetal | |  |
| 6395 | 3 | I | 1 | 2 |  | | ~Birth | |  |
| 6395 | 3 | I | 1 | 3 |  | | <5 Months | |  |
| 6396 | 6 | II | 1 | 1 | Male | | Birth | |  |
| 6396 | 6 | II | 1 | 2 |  | | 1 to 2 Months | |  |
| 6397 | 2 | I | 1 | 1 |  | | ~Birth | |  |
| 6398 | 2 | I | 1 | 1 | Indeterminate | | ≤Birth | | Absent |
| 6398 | 2 | I | 1 | 2 |  | | 2 to 3 Months | |  |
| 6398 | 2 | I | 1 | 3 |  | | 10 to 11 Months | |  |
| 6399 | 1 | I | 1 | 1 |  | | ≤ Birth | | Present |

Key: * = Basket Number.
