# Supplementary material for: Skeletal Remains from Punic Carthage Do Not Support Systematic Sacrifice of Infants
Source: PLoS One. 2010 Feb 17;5(2):e9177. doi: 10.1371/journal.pone.0009177 (PMC2822869; doi:10.1371/journal.pone.0009177)
Supplement: Table S3 — Dimensions of Human Cranial Bones (in mm.), Carthaginian Tophet. (0.33 MB DOC) [file pone.0009177.s003.doc]

| **Urn** | **Individual** | **Pars Basilaris**  **Length** | **Pars Basilaris Width** | | **Hypophyseal Fossa Length** | **Hypophyseal Fossa Width** | **Petrosal**  **Length** | **Side** | **Petrosal Width** | **Side** | |
| --- | --- | --- | --- | --- | --- | --- | --- | --- | --- | --- | --- |
| *2 | 1 | 10.60 | 15.35 | |  |  |  |  | 18.20 | L | |
| *4 | 1 | 13.65 |  | |  |  | 34.40 | R | 15.90 | R | |
| *7 | 1 |  |  | |  |  | 37.30 | L | 15.30 | L | |
| *21 | 1 |  |  | |  |  |  |  | 18.80 | R | |
| *36 | 1 | 11.85 | 15.20 | |  |  |  |  |  |  | |
| *36 | 1 | 11.85 | 15.20 | |  |  |  |  |  |  | |
| *46 | 1 | 11.55 | 12.85 | |  |  |  |  |  |  | |
| *131 | 1 |  |  | |  |  |  |  | 17.40 | L | |
| 2846 | 1 | 11.55 |  | |  |  |  |  |  |  | |
| 3092 | 1 | 12.80 | 14.35 | | 11.35 |  |  |  |  |  | |
| 3093 | 2 |  |  | |  | 18.00 |  |  |  |  | |
| 3160 | 1 |  |  | |  | 20.35 |  |  |  |  | |
| 3163 | 1 | 11.55 |  | |  |  |  |  |  |  | |
| 3164 | 1 | 10.85 | 12.20 | |  |  |  |  |  |  | |
| 3164 | 2 | 12.00 | 15.00 | |  |  |  |  |  |  | |
| 3168 | 1 | 12.80 | 15.00 | | 9.00 | 18.00 |  |  |  |  | |
| 3171 | 1 | 11.80 | 15.05 | | 12.15 | 18.00 |  |  |  |  | |
| 3176 | 1 |  |  | |  | 17.40 |  |  |  |  | |
| 3177 | 1 | 12.20 |  | |  |  |  |  |  |  | |
| 3177 | 1 | 12.20 |  | |  |  |  |  |  |  | |
| 3179 | 1 | 13.00 |  | |  |  |  |  |  |  | |
| 3180 | 1 | 11.90 |  | |  |  |  |  |  |  | |
| 3180 | 1 | 11.90 |  | |  |  |  |  |  |  | |
| 3191 | 1 | 11.00 |  | |  |  |  |  |  |  | |
| 4438 | 1 | 11.90 | 14.20 | |  |  | 31.10 | L | 15.25 | L | |
| 4612 | 1 |  |  | |  |  |  |  | 14.10 | R | |
| 4612 | 1 |  |  | |  |  | 31.90 | R | 16.30 | R | |
| 4941 | 1 | 12.65 | 16.10 | |  |  |  |  |  |  | |
| 4957 | 1 |  |  | |  |  | 28.40 | R | 13.10 | R | |
| 4957 | 2 |  |  | |  |  | 29.10 | R | 13.70 | R | |
| 4957 | 3 |  | |  |  |  | 30.10 | R | 14.90 | R |  |
| 4957 | 4 |  | |  |  |  | 30.60 | R | 15.50 | R |  |
| 5172 | 1 |  | |  |  |  | 36.40 | R | 14.80 | R |  |
| 5173 | 1 | 10.80 | |  |  |  |  |  | 16.80 | R |  |
| 5191 | 1 |  | |  |  |  |  |  | 15.10 | L |  |
| 5409 | 1 |  | |  |  |  | 32.00 | R | 14.70 | R |  |
| 5410 | 1 |  | |  |  |  |  |  | 14.20 | L |  |
| 5412 | 1 |  | |  |  |  | 37.70 | R | 15.50 | R |  |
| 5414 | 1 |  | |  |  |  | 32.20 | R | 12.00 | R |  |
| 5415 | 1 |  | |  |  |  |  |  | 14.70 | L |  |
| 5416 | 1 | 11.10 | | 13.70 |  |  | 29.90 | R | 14.00 | R |  |
| 5417 | 1 |  | |  |  |  | 32.60 | L | 13.20 | L |  |
| 5417 | 1 |  | |  |  |  | 32.60 | L | 13.20 | L |  |
| 5516 | 1 | 10.95 | | 14.15 |  |  | 33.20 | R | 15.10 | R |  |
| 5517 | 1 |  | |  |  |  |  |  | 14.40 | R |  |
| 5517 | 2 |  | |  |  |  | 30.50 | R | 15.50 | R |  |
| 5528 | 1 |  | |  |  |  |  |  | 15.10 | L |  |
| 5529 | 1 |  | |  |  |  | 29.30 | L | 14.50 | R |  |
| 5529 | 2 |  | |  |  |  | 31.80 | R | 16.00 | R |  |
| 5531 | 1 | 12.30 | | 14.00 |  |  | 33.40 | R | 15.40 | R |  |
| 5533 | 1 | 10.90 | | 14.75 |  |  |  |  | 15.70 | R |  |
| 5541 | 1 |  | |  |  |  |  |  | 13.40 | R |  |
| 5541 | 2 | 11.65 | | 13.55 |  | 17.00 | 35.70 | R |  |  |  |
| 5542 | 1 |  | |  |  |  | 36.80 | R | 15.00 | R |  |
| 5544 | 1 |  | |  |  |  | 27.70 | L | 12.20 | L |  |
| 5547 | 1 | 11.80 | |  |  |  |  |  |  |  |  |
| 5550 | 1 |  | |  |  |  |  |  | 15.60 | R |  |
| 5551 | 1 |  | |  |  |  |  |  | 16.40 | R |  |
| 5552 | 1 | 12.25 | |  |  |  |  |  |  |  |  |
| 5553 | 1 |  | |  | 10.40 | 16.50 | 30.20 | R | 15.80 | R |  |
| 5555 | 1 |  | |  |  |  |  |  | 14.40 | L |  |
| 5559 | 1 | 11.50 | | 13.20 |  |  |  |  |  |  |  |
| 5563 | 1 | 10.85 | |  |  |  | 33.90 | L | 15.10 | L |  |
| 5567 | 1 |  | |  |  |  |  |  | 14.10 | R |  |
| 5571 | 1 |  | |  |  |  |  |  | 13.40 | L |  |
| 5574 | 1 | 13.70 | | 22.15 |  |  | 46.70 | R | 21.70 | R |  |
| 5577 | 1 |  | |  | 12.20 | 16.00 | 31.10 | R | 13.20 | R |  |
| 5579 | 1 | 12.50 | | 17.00 |  |  |  |  |  |  |  |
| 5581 | 1 | 11.60 | |  |  |  |  |  | 18.10 | R |  |
| 5582 | 1 | 12.10 | |  | 12.25 | 17.00 |  |  | 14.10 | L |  |
| 5586 | 1 |  | |  |  |  |  |  | 13.00 | L |  |
| 5586 | 2 |  | |  |  |  | 45.60 | L |  |  |  |
| 5587 | 1 |  | |  |  |  |  |  | 14.40 | L |  |
| 5588 | 1 |  | |  |  | 17.65 |  |  | 15.90 | R |  |
| 5590 | 1 | 12.05 | | 14.15 |  |  |  |  |  |  |  |
| 5591 | 1 |  | |  |  |  |  |  | 17.50 | L |  |
| 5592 | 1 |  | |  |  |  | 24.80 | L | 12.70 | L |  |
| 5592 | 2 |  | |  |  |  |  |  | 13.70 | L |  |
| 5595 | 1 | 12.25 | | 14.30 |  |  | 37.70 | R | 14.10 | R |  |
| 5597 | 1 |  | |  |  |  |  |  | 14.20 | L |  |
| 5599 | 1 | 11.95 | | 14.40 |  |  |  |  | 15.30 | R |  |
| 5603 | 1 |  | |  |  |  |  |  | 15.10 | R |  |
| 5604 | 1 | 13.10 | |  |  |  |  |  |  |  |  |
| 5623 | 1 |  | |  | 11.60 | 16.45 |  |  | 13.50 | R |  |
| 5647 | 1 |  | |  |  |  |  |  | 14.60 | R |  |
| 5816 | 1 |  | |  |  |  |  |  | 17.20 | R |  |
| 5821 | 1 | 10.65 | |  |  |  |  |  |  |  |  |
| 5824 | 1 | 10.80 | | 12.65 | 10.00 | 18.70 | 38.30 | R | 16.20 | R |  |
| 5827 | 1 | 12.55 | |  |  |  |  |  |  |  |  |
| 5834 | 1 | 13.00 | | 13.10 |  | 16.60 | 34.70 | R | 13.10 | R |  |
| 5835 | 1 | 12.15 | | 14.90 | 11.50 | 17.45 | 31.95 | L | 16.50 | L |  |
| 5838 | 1 | 8.95 | | 12.00 | 9.40 |  | 29.10 | R | 14.40 | R |  |
| 5839 | 1 |  | |  |  |  |  |  | 17.60 | R |  |
| 5840 | 1 |  | |  |  |  |  |  | 16.20 | R |  |
| 5841 | 1 |  | |  |  |  |  |  | 13.80 | R |  |
| 5842 | 1 |  | |  |  |  | 34.40 | R | 15.20 | L |  |
| 5843 | 1 |  | |  |  |  |  |  | 15.10 | L |  |
| 5844 | 1 |  | |  |  |  |  |  | 16.10 | R |  |
| 5850 | 1 | 11.35 | |  |  |  |  |  |  |  |  |
| 5854 | 1 | 10.40 | | 14.05 |  |  |  |  | 15.50 | L |  |
| 5861 | 1 |  | |  |  |  | 32.90 | L | 15.10 | L |  |
| 5867 | 1 | 11.60 | | 13.95 | 11.40 | 16.75 | 33.10 | R | 13.40 | R |  |
| 5874 | 1 |  | |  |  |  | 38.40 | L | 17.40 | L |  |
| 5875 | 1 | 12.25 | | 14.40 |  | 19.40 |  |  | 15.10 | L |  |
| 5876 | 1 |  | |  |  |  |  |  | 20.80 | L |  |
| 5880 | 1 | 11.90 | | 15.05 |  |  |  |  | 15.90 | L |  |
| 5882 | 1 | 10.25 | |  | 11.00 | 17.00 |  |  |  |  |  |
| 5883 | 1 | 10.05 | |  |  |  | 34.90 | R | 17.10 | R |  |
| 5884 | 1 | 12.15 | | 15.60 |  | 19.50 | 37.10 | R | 15.70 | R |  |
| 5885 | 1 |  | |  |  |  |  |  | 15.10 | L |  |
| 5886 | 1 | 11.70 | |  |  |  |  |  |  |  |  |
| 5887 | 1 | 11.60 | | 13.30 |  |  | 32.90 | R | 14.10 | R |  |
| 5887 | 2 |  | |  |  |  |  |  | 14.70 | R |  |
| 5888 | 1 |  | |  | 13.00 | 17.40 | 35.20 | L | 16.90 | L |  |
| 5890 | 1 |  | |  |  |  | 35.50 | L | 17.10 | L |  |
| 5891 | 1 |  | |  |  |  | 37.90 | R | 17.70 | R |  |
| 5892 | 1 | 11.90 | |  |  | 17.10 |  |  |  |  |  |
| 5893 | 1 |  | |  |  |  |  |  | 14.90 | L |  |
| 5894 | 1 | 11.80 | | 14.00 | 10.00 | 16.75 |  |  |  |  |  |
| 5895 | 1 |  | |  |  |  |  |  | 16.30 | L |  |
| 5897 | 1 |  | |  |  |  | 36.80 | L | 16.80 | L |  |
| 5899 | 1 |  | |  |  |  | 31.20 | R | 14.40 | R |  |
| 5902 | 1 | 13.05 | | 15.75 |  |  | 32.70 | R | 19.60 | R |  |
| 5903 | 1 |  | |  |  |  | 28.90 | R | 14.40 | R |  |
| 5904 | 1 |  | |  |  |  |  |  | 19.60 | L |  |
| 5920 | 1 |  | |  |  |  |  |  | 13.20 | L |  |
| 5920 | 2 |  | |  |  |  | 36.00 | R | 15.70 | R |  |
| 5923 | 1 |  | |  |  |  | 30.40 | L | 12.60 | L |  |
| 5923 | 2 |  | |  |  |  |  |  | 16.10 | R |  |
| 5927 | 1 | 11.60 | | 13.85 |  |  | 31.10 | R | 12.40 | R |  |
| 5928 | 1 |  | |  |  |  | 33.02 | L | 14.10 | L |  |
| 5929 | 1 | 13.65 | | 19.60 |  |  |  |  | 14.40 | R |  |
| 5930 | 1 |  | |  |  |  |  |  | 15.00 | R |  |
| 5931 | 1 |  | |  |  |  | 30.10 | L | 14.50 | L |  |
| 5932 | 1 |  | |  |  |  | 40.30 | R | 14.90 | R |  |
| 5934 | 1 |  | |  |  |  | 41.30 | L | 15.80 | L |  |
| 5938 | 1 |  | |  |  |  | 35.00 | R | 13.10 | R |  |
| 5939 | 1 | 11.80 | | 14.40 |  |  | 35.00 | L | 14.70 | L |  |
| 5941 | 1 |  | |  |  |  |  |  | 14.60 | R |  |
| 5944 | 1 |  | |  |  |  |  |  | 14.60 | R |  |
| 5945 | 1 | 12.60 | | 15.30 | 12.20 | 19.00 | 35.20 | R | 14.95 | R |  |
| 5946 | 1 | 13.80 | | 14.90 |  |  | 31.40 | L | 12.40 | L |  |
| 5948 | 1 |  | |  |  |  |  |  | 15.80 | R |  |
| 5950 | 1 | 11.60 | |  |  | 18.55 | 31.10 | L |  |  |  |
| 5952 | 1 |  | |  |  |  | 22.70 | L | 12.30 | L |  |
| 5954 | 1 |  | |  |  |  |  |  | 15.10 | R |  |
| 5959 | 1 |  | |  | 9.00 | 16.70 | 35.90 | R | 16.10 | R |  |
| 5959 | 2 |  | |  |  |  |  |  | 17.90 | R |  |
| 5963 | 1 |  | |  |  | 18.00 |  |  |  |  |  |
| 5965 | 1 | 11.50 | |  |  |  |  |  |  |  |  |
| 5966 | 1 | 11.60 | | 13.85 |  |  |  |  | 15.30 | L |  |
| 5967 | 1 |  | |  |  |  |  |  | 15.60 | R |  |
| 5968 | 1 |  | |  |  |  | 33.60 | R | 16.90 | R |  |
| 5970 | 1 |  | |  |  |  | 31.90 | R | 18.80 | R |  |
| 5971 | 1 |  | |  |  |  | 34.90 | R | 14.90 | R |  |
| 5973 | 1 |  | |  |  |  | 44.40 | R | 19.60 | R |  |
| 5982 | 1 |  | |  |  | 18.30 |  |  | 15.60 | R |  |
| 5984 | 2 |  | |  |  |  |  |  | 17.40 | R |  |
| 5986 | 1 | 11.95 | | 15.25 | 13.50 | 18.60 | 39.20 | R | 17.10 | R |  |
| 5986 | 2 |  | |  |  |  |  |  | 17.30 | R |  |
| 5987 | 1 | 14.90 | |  |  |  |  |  |  |  |  |
| 5989 | 1 |  | |  |  |  |  |  | 13.30 | L |  |
| 5991 | 1 |  | |  |  | 19.00 |  |  | 15.80 | L |  |
| 5992 | 1 |  | |  |  | 13.45 | 35.90 | R | 15.70 | R |  |
| 5995 | 1 | 12.60 | | 14.20 |  |  | 31.30 | L | 14.70 | L |  |
| 5995 | 2 |  | |  |  |  |  |  | 14.00 | L |  |
| 5997 | 1 |  | |  |  |  |  |  | 15.50 | L |  |
| 5998 | 1 |  | |  |  |  |  |  | 14.80 | L |  |
| 6000 | 1 | 11.70 | |  |  |  | 38.20 | L | 14.10 | L |  |
| 6005 | 1 |  | | 19.75 |  |  |  |  | 16.10 | L |  |
| 6006 | 1 | 12.05 | | 13.20 |  |  |  |  |  |  |  |
| 6024 | 1 | 11.51 | | 13.50 |  |  | 29.10 | L | 13.60 | L |  |
| 6024 | 2 |  | |  |  |  | 33.30 | L | 15.00 | L |  |
| 6026 | 1 |  | |  | 12.00 | 19.00 |  |  |  |  |  |
| 6027 | 1 |  | |  | 13.00 | 16.50 | 32.20 | L | 16.10 | L |  |
| 6028 | 1 |  | |  |  |  | 22.00 | L | 12.00 | L |  |
| 6028 | 2 |  | |  |  |  | 26.60 | L | 13.90 | L |  |
| 6028 | 3 | 12.70 | | 14.32 | 10.60 | 17.70 | 35.00 | R | 14.80 | R |  |
| 6029 | 1 |  | |  |  |  |  |  | 13.35 | L |  |
| 6029 | 2 |  | |  |  |  | 33.90 | L | 13.65 | L |  |
| 6029 | 3 |  | |  |  |  | 33.80 | R | 14.00 | L |  |
| 6030 | 1 |  | |  | 13.30 |  | 32.20 | L | 15.80 | L |  |
| 6031 | 1 |  | |  | 11.10 | 19.75 |  |  |  |  |  |
| 6031 | 2 |  | |  | 11.15 | 15.65 |  |  |  |  |  |
| 6033 | 1 |  | |  |  |  | 36.20 | R | 16.80 | R |  |
| 6034 | 1 |  | |  |  |  |  |  | 15.80 | R |  |
| 6035 | 1 | 13.40 | | 15.50 |  |  |  |  | 15.50 | R |  |
| 6036 | 1 |  | |  |  |  | 30.60 | L | 12.30 | L |  |
| 6037 | 1 | 12.41 | | 15.20 | 12.00 | 18.30 |  |  | 15.30 | R |  |
| 6038 | 1 |  | |  |  |  | 28.90 | R | 13.20 | R |  |
| 6039 | 1 |  | |  |  |  | 36.90 | L | 15.20 | L |  |
| 6042 | 1 |  | |  |  |  |  |  | 15.50 | L |  |
| 6044 | 1 |  | |  |  |  |  |  | 17.40 | R |  |
| 6047 | 1 | 13.30 | | 15.60 | 12.30 | 19.80 | 34.90 | R | 16.50 | R |  |
| 6049 | 1 |  | | 14.25 |  |  |  |  | 15.90 | R |  |
| 6050 | 1 | 11.95 | | 13.60 |  |  |  |  | 15.10 | R |  |
| 6052 | 1 | 12.55 | | 15.05 |  |  | 39.90 | L | 17.80 | L |  |
| 6053 | 1 |  | |  |  |  |  |  | 15.20 | R |  |
| 6054 | 1 |  | |  |  |  | 34.10 | L | 13.20 | L |  |
| 6055 | 1 | 10.15 | | 13.05 |  |  |  |  |  |  |  |
| 6062 | 1 | 11.00 | | 14.65 |  | 17.25 | 37.10 | R | 13.20 | R |  |
| 6064 | 1 |  | |  |  |  |  |  | 14.40 | R |  |
| 6066 | 1 |  | |  |  |  |  |  | 15.20 | R |  |
| 6067 | 1 | 13.50 | | 16.30 |  |  | 35.10 | L | 15.30 | L |  |
| 6068 | 1 | 11.60 | | 13.45 |  |  | 36.20 | R | 14.40 | R |  |
| 6069 | 1 |  | |  |  |  | 35.80 | L | 16.00 | L |  |
| 6071 | 1 |  | |  | 11.40 | 16.75 |  |  | 14.80 | R |  |
| 6073 | 1 |  | |  |  |  |  |  | 16.30 | R |  |
| 6075 | 1 |  | |  |  |  | 29.90 | R | 13.90 | R |  |
| 6076 | 1 |  | |  |  |  | 26.80 | L | 14.40 | L |  |
| 6077 | 1 | 11.25 | | 13.75 |  |  |  |  | 15.80 | L |  |
| 6081 | 1 | 12.60 | | 16.25 |  |  | 35.80 | L | 14.90 | L |  |
| 6082 | 1 | 11.80 | | 14.60 |  |  |  |  | 14.40 | R |  |
| 6082 | 2 | 11.45 | | 15.55 |  |  | 29.90 | R | 15.10 | R |  |
| 6111 | 1 |  | |  |  |  |  |  | 13.90 | R |  |
| 6379 | 1 |  | |  |  | 17.50 | 38.10 | L | 19.50 | L |  |
| 6380 | 1 |  | |  |  |  | 34.60 | R | 16.00 | R |  |
| 6386 | 1 | 12.00 | | 14.30 |  |  | 30.40 | L | 15.40 | L |  |
| 6388 | 1 |  | |  |  |  | 33.90 | L | 15.50 | L |  |
| 6392 | 1 | 10.45 | | 14.30 |  |  |  |  |  |  |  |
| 6392 | 2 | 11.60 | | 15.65 |  |  |  |  | 15.30 | L |  |
| 6394 | 1 | 11.30 | | 14.50 | 11.20 | 18.30 |  |  | 14.50 | R |  |
| 6395 | 2 | 11.50 | |  |  |  | 39.03 | L | 17.10 | L |  |

Key: * = Basket Number; R = Right; L = Left
