# Supplementary material for: Skeletal Remains from Punic Carthage Do Not Support Systematic Sacrifice of Infants
Source: PLoS One. 2010 Feb 17;5(2):e9177. doi: 10.1371/journal.pone.0009177 (PMC2822869; doi:10.1371/journal.pone.0009177)
Supplement: Table S4 — Dimensions of Human Pelvic Bones (in mm.), Carthaginian Tophet. (0.12 MB DOC) [file pone.0009177.s004.doc]

| **Urn** | **Individual** | **Ischium Length** | **Side** | **Ischium Height** | **Side** | **Pubis Length** | **Side** |
| --- | --- | --- | --- | --- | --- | --- | --- |
| *9 | 1 | 17.20 | R | 12.00 | R |  |  |
| *21 | 1 |  |  |  |  | 14.45 | L |
| *168 | 1 | 21.75 | R |  |  |  |  |
| 2846 | 1 | 21.00 | L | 14.00 | L |  |  |
| 3093 | 1 | 12.00 | L |  |  |  |  |
| 3093 | 2 | 17.00 | L |  |  |  |  |
| 3164 | 1 | 17.00 | R | 11.00 | R |  |  |
| 3171 | 1 |  |  |  |  | 14.50 | L |
| 3190 | 1 |  |  |  |  | 19.80 | L |
| 3191 | 1 | 17.00 | R | 11.00 | R |  |  |
| 3191 | 2 | 18.30 | R | 11.40 | R |  |  |
| 3193 | 1 | 19.00 | L |  |  |  |  |
| 4438 | 1 | 18.60 | L |  |  |  |  |
| 4614 | 1 |  |  |  |  | 16.55 | L |
| 5090 | 1 | 18.20 | R | 11.05 | R | 15.70 | R |
| 5173 | 1 | 18.70 | R | 12.00 | R | 15.00 | L |
| 5189 | 1 |  |  |  |  | 15.75 | L |
| 5190 | 1 | 17.70 | R | 11.30 | L |  |  |
| 5410 | 1 |  |  |  |  | 16.00 | R |
| 5517 | 2 | 15.50 | L |  |  | 13.60 | R |
| 5529 | 2 | 18.00 | R |  |  |  |  |
| 5546 | 1 |  |  |  |  | 18.00 | L |
| 5552 | 1 |  |  |  |  | 17.00 | L |
| 5552 | 2 |  |  |  |  | 17.20 | L |
| 5577 | 1 |  |  | 12.80 | L | 17.40 | R |
| 5589 | 1 |  |  |  |  | 18.00 | R |
| 5603 | 1 |  |  |  |  | 15.80 | L |
| 5824 | 1 | 19.20 | R | 12.40 | L | 16.40 | R |
| 5827 | 1 |  |  |  |  | 14.40 | L |
| 5829 | 1 | 19.55 | L |  |  |  |  |
| 5835 | 1 | 18.20 | L |  |  | 14.50 | L |
| 5849 | 1 | 23.60 | R |  |  |  |  |
| 5850 | 1 | 21.20 | L | 13.00 | L | 19.30 | R |
| 5854 | 1 | 18.00 | R |  |  |  |  |
| 5869 | 1 |  |  | 12.65 | L | 15.00 | L |
| 5883 | 1 |  |  | 11.50 | L | 14.50 | L |
| 5894 | 1 | 17.40 | R | 11.00 | R | 14.00 | L |
| 5895 | 1 | 21.20 | R | 15.80 | R |  |  |
| 5903 | 1 | 18.40 | R |  |  |  |  |
| 5920 | 1 | 17.40 | R | 12.00 | R |  |  |
| 5920 | 2 | 20.00 | R | 12.00 | R | 16.00 | R |
| 5923 | 2 | 17.45 | R |  |  |  |  |
| 5932 | 1 |  |  | 14.90 | L |  |  |
| 5945 | 1 | 18.65 | R | 12.60 | R |  |  |
| 5948 | 1 | 21.35 | L | 12.75 | L |  |  |
| 5954 | 1 | 18.60 | L |  |  |  |  |
| 5959 | 1 | 18.55 | R | 12.00 | R | 15.00 | R |
| 5963 | 1 | 16.75 | L | 11.80 | L | 19.35 | R |
| 5967 | 1 | 19.45 | R | 11.65 | R |  |  |
| 5986 | 1 | 19.90 | R | 12.90 | R | 18.25 | R |
| 5987 | 1 |  |  |  |  | 23.30 | R |
| 5990 | 1 | 17.30 | L | 11.00 | L |  |  |
| 6000 | 1 | 21.00 | L |  |  | 11.80 | L |
| 6024 | 2 | 17.30 | L | 11.00 | L |  |  |
| 6032 | 1 |  |  |  |  | 15.70 | L |
| 6033 | 1 | 20.00 | R |  |  | 16.00 | R |
| 6036 | 1 | 19.30 | R |  |  |  |  |
| 6043 | 1 | 15.95 | R | 10.45 | R |  |  |
| 6047 | 1 | 19.70 | R | 12.55 | R | 16.20 | R |
| 6052 | 1 |  |  |  |  | 14.40 | R |
| 6053 | 1 |  |  |  |  | 19.95 | R |
| 6062 | 1 | 12.30 | R |  |  |  |  |
| 6071 | 1 |  |  | 11.40 | L |  |  |
| 6386 | 1 |  |  |  |  | 14.65 | R |
| 6387 | 1 |  |  |  |  | 16.15 | L |

Key: * = Basket Number; R = Right; L = Left.
